# Transport of Carbon Dioxide, Methane, Oxygen and Nitrogen in a Glassy Polyimide Membrane

**DOI:** 10.3390/molecules30234524

**Published:** 2025-11-23

**Authors:** Marek Tańczyk, Aleksandra Janusz-Cygan, Anna Pawlaczyk-Kurek, Łukasz Hamryszak, Jolanta Jaschik

**Affiliations:** Institute of Chemical Engineering, Polish Academy of Sciences, ul. Bałtycka 5, 44-100 Gliwice, Poland; ajcygan@iich.gliwice.pl (A.J.-C.); ania.pawlaczyk@iich.gliwice.pl (A.P.-K.); lukasz.hamryszak@iich.gliwice.pl (Ł.H.); jjaschik@iich.gliwice.pl (J.J.)

**Keywords:** biogas separation, polyimide-based membrane, glassy polymer, competitive sorption, Dual Mode Sorption model, partial immobilization model, ideal selectivity

## Abstract

Biomethane is one of the controllable Renewable Energy Sources. It may be derived from biogas, a multicomponent gas mixture, using, among others, membrane processes. The proper optimization of such a process requires the knowledge of the phenomena accompanying each specific biogas–membrane separation system. Therefore, the solubility, permeance and diffusion of CO_2_, CH_4_, O_2_ and N_2_ in a polyimide-based sample were described and analyzed using the Dual Mode Sorption and partial immobilization models. The parameters of the models were determined based on pure gas sorption isotherms measured gravimetrically and experimental permeances of the four gases. The membrane swelling caused by CO_2_ was observed at temperatures of 293 and 303 K and for pressures higher than 3 bar. The adsorption of CH_4_, O_2_ and N_2_ in the fractional free volume (FFV) has a dominant (>50%) share in their total solubility in the entire pressure range. This makes them sensitive to the presence of CO_2_, whose affinity is the strongest towards the tested polyimide-based sample. The diffusion of O_2_ is the fastest which makes it competitive with CO_2_ in permeation through the membrane, despite its low solubility. The ideal CO_2_/O_2_ selectivity is thus relatively low (2.3–5.1). Methane, which is competitive in solubility compared to CO_2_, was found to diffuse the slowest and as a result, it is also the slowest permeating gas. This translates into the very high CO_2_/CH_4_ ideal selectivity (33–95.7), which is, however, strongly dependent on temperature and pressure.

## 1. Introduction

Europe is currently transitioning from fossil fuels to green energy sources. This means that the share of solar and wind energy on the supply side of the energy system is growing. It makes maintaining the stability of the power grid increasingly difficult. However, the energy system should be able to respond flexibly to fluctuations in supply and demand, and in such a situation, the so-called controllable Renewable Energy Sources (RES) play an important role. One of RESs is biomethane, included in the European Green Deal and the associated Methane Emissions Reduction Strategy.

Biomethane is an upgraded biogas with parameters of natural gas, whereas biogas is a gaseous product of methane fermentation of organic compounds. In the case of biogas of agricultural origin, its main components are methane (53–85 vol.%), carbon dioxide (14–48 vol.%), nitrogen (0.5–7.5 vol.%) and oxygen (<1 vol.%) [1,2,3,4]. It may contain water vapor (1–10 vol.%) and H_2_S (10–30,000 ppm) [2], and in the case of municipal waste, also siloxanes. However, before upgrading biogas to biomethane, the raw biogas must be pre-treated, i.e., freed from hydrogen sulfide, solid particles and moisture. Only then is the biogas prepared and can be directed to the membrane, adsorption or absorption installation. The European Biogas Association (EBA) reports that Europe produced 4.2 billion m^3^ of biomethane in 2022, with a target of 35 billion m^3^ by 2030 [5].

In order to upgrade biogas to biomethane at high throughput (several hundred or more m^3^ h^−1^ of biogas), technologies based on membrane and adsorptive separation processes are already used [6,7,8,9,10]. Nevertheless, extensive research and development efforts are currently ongoing, with a focus on the identification of novel processes that would facilitate the separation of biogas into two valuable streams: bio-CH_4_ and bio-CO_2_. These processes should be characterized by high purity and recovery of both products as well as good energy efficiency. In this case, technologies for the removal of carbon dioxide from gas streams can be used, e.g., a hybrid VSA-membrane installation [11]. A high-purity CO_2_ stream is produced in the second, membrane stage of this installation, to which a mixture containing 50–70 vol.% of carbon dioxide is fed at a pressure of 3–10 bar. This stage uses commercially available polysulfone- and polyimide-based membranes and should be optimized for biogas upgrading.

The simulation of membrane gas separation processes using physicochemical models requires knowledge of parameters characterizing the mass transport of mixture components through the membrane. The most commonly used mass transfer coefficient in this case is a permeance (or permeability when the membrane thickness is known). It can be determined based on the directly measured permeate flow and the driving force of the process (usually the difference in the partial pressure of the permeating gas). For modeling and simulation purposes, it can be assumed in some cases that the components of the gas mixture penetrate the membrane independently. In such a situation, permeances determined for pure gases are sufficient to describe the mass transport through the membrane. They can be relatively easily determined using standard experimental techniques or found in the already large literature database. This approach ensures good agreement between numerical simulations and experimental data in the case of separation of CO_2_/N_2_ mixtures, e.g., in the process of CO_2_ removal from exhaust gases [11,12,13,14,15]. However, this ideal selectivity, based on the permeance of pure gases (cf. Equation (2)) is often overestimated, due to phenomena such as competitive sorption, plasticization, swelling or the non-ideality of gases [16]. In the case of mixtures containing CH_4_ and CO_2_, competitive sorption takes place in the fractional free volume (FFV) of a glassy polymer that forms the active layer of the membrane [17,18,19]. Moreover, in many membrane materials the CO_2_ accelerated plasticization takes place [20,21,22]. The co-occurrence of both these phenomena (competitive sorption and plasticization) is thus the most often reported as a reason for deviation from the ideal pure-gas selectivity, when CO_2_ and CH_4_ permeate through the membrane together [22,23,24]. In practical applications, plasticization should be avoided. However, the mere presence of CO_2_, which pushes polymer chains apart, can facilitate the transport of other components of the gas mixture (including methane) within the membrane matrix [25,26,27]. Conversely, the presence of very slowly diffusing CH_4_ hinders the diffusion of CO_2_ within the polymer matrix, as reported in [19,28,29].

In addition to the phenomena mentioned above, it should be taken into account that the biogas sent for separation may also contain oxygen and nitrogen. Moreover, permeance coefficients in polymer membranes are temperature dependent [30,31]. Taking all this into account there is a lack of relevant data for the specific membrane materials used in a separation process which is designed or being optimized.

In the previous work [19], the solubility and diffusion of carbon dioxide and methane in pure and mixed states were analyzed in a polysulfone-based membrane sample from the Air Products PRISM PA1020–P1 module. Due to the range of available data, this analysis was limited to one temperature (295 K). In this work, the phenomena accompanying the permeation, solubility and diffusion of the four pure biogas components in a polyimide-based membrane sample were investigated in the temperature range of 293–328 K.

Hence, the sorption of carbon dioxide, methane, oxygen and nitrogen in the polyimide-based membrane from the commercially available UBE UMS-A2 module was investigated experimentally in the pressure and temperature range in which the plasticization or the non-ideality of gases could be avoided. The Dual Mode Sorption (DMS) model [29] was used to describe and analyze the solubility of these gases in the studied glassy polymer membrane and to assess their competitiveness.

Moreover, individual permeances of these four biogas components were determined in the UBE UMS-A2 module as a function of temperature and pressure. Based on them and using experimental solubilities, the diffusivities of CO_2_, CH_4_, O_2_ and N_2_ in the membrane material from this module, as well as their dependence on pressure and temperature, were determined. The use of the partial immobilization model [29] allowed the identification and explanation of the diffusion phenomena accompanying the permeation of the main biogas components in the investigated glassy polymer membrane.

## 2. Results and Discussion

In order to describe the mass transfer of a gas component i-th in a dense polymeric membrane the solution-diffusion model is usually used [22]. It is assumed in the model that this component dissolves and diffuses in the polymer structure, and furthermore, the product of solubility (S_i_) and diffusion coefficient (D_i_) provides permeability (P_i_), which serves as the overall mass transfer coefficient in membrane processes:

(1) $$P_{i}=S_{i}\cdot D_{i}=Q_{i}\cdot \delta_{M}$$

The permeability can be related to the permeance (Q_i_) and membrane thickness (δ_M_), as was shown in the above equation. The permeance can be directly derived from the mass flux permeating through the membrane and the pressure difference on both of its sides, measured in permeation experiments [3,21].

The basic parameter describing the separation performance of a membrane material is a permselectivity (α_i/j_), defined as the ratio between the permeability coefficients of two gases, i-th and j-th. Taking into account the solution-diffusion model, the permselectivity of component i-th vs. component j-th may be expressed as a product of solubility-selectivity (α_(S)i/j_) and diffusivity-selectivity (α_(D)i/j_):

(2) $$\propto_{i/j}=\frac{P_{i}}{P_{j}}=\left(\frac{S_{i}}{S_{j}}\right)\cdot \left(\frac{D_{i}}{D_{j}}\right)=\propto_{\left(S\right)i/j}\cdot \propto_{\left(D\right)i/j}$$

If parameters in Equation (2) refer to pure gases, then one speaks of ideal selectivity. The solubility of gaseous species i-th in Equations (1) and (2) is expressed as:

(3) $$S_{i}=\frac{C_{i}}{p_{i}}$$

where C is the gas concentration in a polymer (cm^3^ (STP) cm^−3^) and p is the gas pressure (bar).

### 2.1. Sorption of CO_2_, CH_4_, O_2_ and N_2_

The concentration of pure CO_2_, CH_4_, O_2_ and N_2_ in the polyimide-based membrane from the UBE UMS-A2 module was measured experimentally using the gravimetric analyzer and methodology described in Section 3.3. The appropriate isotherms were determined at temperatures in the range of 293–328 K and pressures in the range of 0–18 bar. At the given temperature, the sorption points were first measured when the pressure was increased step by step from 0 to the maximum. In the second step, the desorption points were measured when the pressure was decreased in the same way from the maximum pressure to 0.

**Figure 1:**
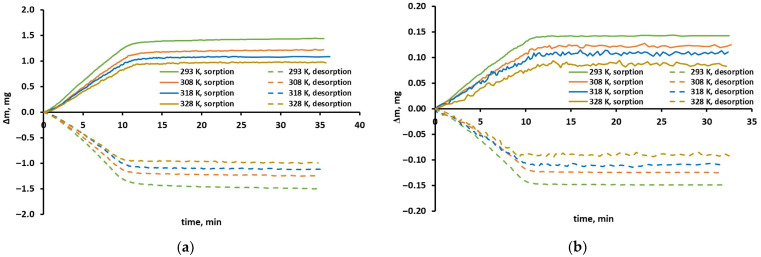
Sample mass uptake curves of pure (**a**) CO_2_ and (**b**) CH_4_ in the polyimide-based membrane from the UBE UMS-A2 module registered during pressure change from 3 to 5 bar (sorption) and 5 to 3 bar (desorption).

**Figure 2:**
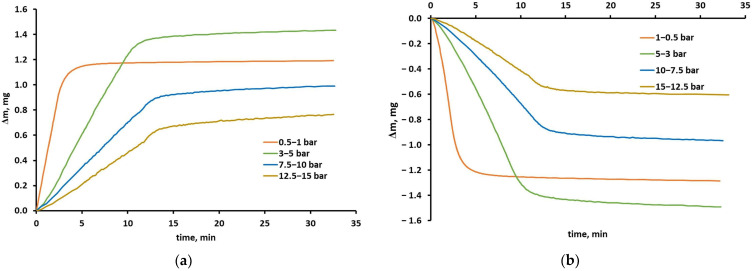
Mass uptake curves registered at 293 K during (**a**) sorption and (**b**) desorption of pure CO_2_ in the polyimide-based membrane from the UBE UMS-A2 module at different pressure levels.

**Figure 3:**
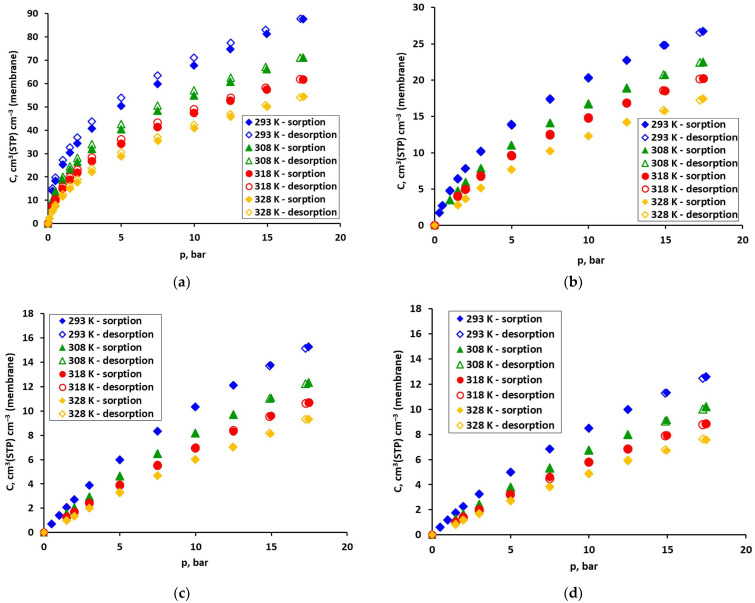
Experimental sorption/desorption isotherms of pure (**a**) CO_2_, (**b**) CH_4_, (**c**) O_2_, and (**d**) N_2_, in the polyimide-based membrane sample from the UBE UMS-A2 module.

In the case of every isotherm point the change in the sample mass over time, illustrating the sorption/desorption of gas due to the change in its pressure was registered and monitored. This dependence of mass change on time is hereinafter referred to as the sorption/mass uptake curve. As an example, it is shown in Figure 1 for the sorption and desorption of CO_2_ and CH_4_ in the pressure range of 3–5 bar and in Figure 2 for CO_2_ at 293 K. The sorption/desorption isotherms are shown in Figure 3; however, before their analysis, the phenomena that can be observed during the determination of individual points of these isotherms are discussed directly below. In the case shown in Figure 1, the full mass uptake was reached in about 10–12 min. In the case of methane sorption and desorption, the sample weight did not change after this time. The same situation occurred in the case of the sorption of carbon dioxide. This means that the equilibrium was achieved. In the case of CO_2_ desorption, the sample mass decreases slightly but systematically, and equilibrium is not reached within the maximum time assumed for measuring one isotherm point. In fact, this monotonic change in sample weight over time accompanies not only the desorption of CO_2_ at other pressure levels, but also occurs during the sorption of this gas at higher pressures. This is illustrated more clearly in Figure 2. The consequence of this phenomenon is the appearance of hysteresis in the case of carbon dioxide isotherms, which will be discussed later. It should only be noted at this point that the shape of the sorption curves in Figure 1 and Figure 2 suggests that equilibrium is established very quickly. The change in sample weight during the first 10–13 min practically follows the change in pressure over time and only a short, gentle transition between the rising and horizontal sections indicates a limited rate of mass transport. Thus, each line in Figure 1 is associated with the corresponding isotherm point in Figure 3a,b for a pressure of 5 bar (sorption isotherm) or 3 bar (desorption isotherm).

In the cases presented in Figure 1, the changes in the sample weight amount to 960–1520 µg and 84–150 µg for carbon dioxide and methane, respectively, and they significantly exceed the measurement resolution of the microbalance (0.2 μg). On the methane sorption curves, one can observe sample weight fluctuations. They are more pronounced the higher the temperature, and therefore the smaller the sample weight change. In fact, these fluctuations accompany each uptake curve. This is due to the fact that the key parameters, temperature and pressure, are stabilized with a high but limited accuracy of ±0.2 K and ±0.02% of the measurement range, respectively. Therefore, the microbalance with its high weighting resolution of ±0.2 μg will record even a very small mass change associated with temperature and pressure fluctuations.

The experimental isotherms of carbon dioxide, methane, oxygen and nitrogen are shown in Figure 3. As can be seen in these figures, the sorption capacity of the polyimide membrane sample is the highest for CO_2_, followed by methane, oxygen and nitrogen. The isotherm of carbon dioxide is strongly nonlinear. The similar concentrations of carbon dioxide and methane in other polyimide-based materials are presented in [27,31,32,33,34]. According to the data presented there, at 308 K and 15 bar, the solubility of CO_2_ is in the range of 40–120 cm^3^ (STP) cm^−3^ (membrane), and the solubility of CH_4_ is from 10 to 55 cm^3^ (STP) cm^−3^ (membrane). The solubility of nitrogen (2–8 cm^3^ (STP) cm^−3^ at 308 K and 15 bar) in polyimide-based membranes has been reported in the works [31,32,34]. Only Wang’s group determined the solubility of oxygen (10 cm^3^ (STP) cm^−3^ at 308 K and 15 bar) in a polyimide 6FDA-6FpDA membrane [32].

As can be seen in Figure 3, the sorption and desorption points of the CH_4_, O_2_ and N_2_ isotherms overlap over the entire pressure range. In these cases, the sample always returned to its original state after regeneration. This means that it retained its initial weight and sorption capacity. These gases do not cause any observable and permanent changes in the membrane material; therefore, their sorption is a completely reversible process. The details of sample regeneration are given in Section 3.3.

In the case of carbon dioxide, the desorption points are located above the sorption points. This would mean that no equilibrium was reached during desorption or during both sorption and desorption. However, the measurement time of a single isotherm point seems to have been long enough, as it was discussed earlier. Hysteresis is therefore observed. One can assume that the hysteresis indicates the occurrence of additional phenomena accompanying physical sorption/desorption. Some evidence indicates that one of these phenomena is CO_2_ condensation in FFV. The desorption is then hindered and may be incomplete if time is limited. This can actually be seen in Figure 1a and Figure 2b. In such a situation the desorption points are located higher than the sorption points on the isotherm graph, as can be seen in Figure 3a. Furthermore, the tendency of gas/vapor to condense is stronger at lower temperatures and higher pressures. Indeed, as can be seen in Figure 3a, the hysteresis is most pronounced at 293 K, while at 328 K the sorption and desorption points almost coincide. Confirmation of this can also be found in Figure 1a by comparing the absolute values of the sample mass change (Δm) during sorption and desorption in the same pressure range of 3–5 bar. At 328 K, these values are almost the same and are 0.96 mg and 0.99 mg for sorption and desorption at the end of the measurement, respectively.

Therefore, the monotonic, practically linear decrease in sample weight after pressure stabilization visible in desorption curves indicates more difficult desorption of condensed CO_2_. The shape of the CO_2_ sorption curves depends on pressure and temperature. As can be seen in Figure 2a (orange line), for pressures below 3 bar and at a temperature of 308 K, the sample weight does not change, and the sorption curve remains parallel to the abscissa axis until the end of measurement of a given isotherm point. Under these conditions, condensation probably does not occur, which was also discussed in our previous work on CO_2_ sorption in a polysulfone-based sample [19]. The lack of change in the sample weight after reaching equilibrium can also be observed in the case of sorption curves recorded in the pressure range of 3–5 bar, at temperatures of 318 and 328 K. However, under these conditions, condensation does occur, as evidenced by the appearance of hysteresis (Figure 3a) and the shape of the desorption curves (Figure 1a and Figure 2b). This image is typical in the case of physical adsorption of gases or vapors in solids with a stable micropore (voids) structure. The empty volume for the condensing gas is then fixed and stable.

However, in the case of sorption curves recorded at temperatures of 293 and 303 K (Figure 1a and Figure 2a) and pressures higher than 3 bar (Figure 2a), a monotonic, practically linear increase in the sample weight can be observed after the pressure stabilizes. This shape of the sorption curves most likely indicates a constant increase in the sorption volume of the tested polyimide sample due to a parallel and progressive condensation of CO_2_. Such a phenomenon is identified in the literature as a swelling [35,36]. It intensifies when the pressure is increased and the temperature is decreased, i.e., under the same conditions that simultaneously promote the condensation of carbon dioxide in the FFV of the glassy polymer. In the current study, the occurrence of this phenomenon can be observed even at a relatively low CO_2_ pressure (above 3 bar at 293 K). A similar situation occurred in the case of the polysulfone-based sample investigated in our previous work [19], although this material seems to be slightly more resistant to swelling, which at a temperature of 293 K was observed only at a pressure higher than 5 bar.

The swelling of a membrane material is unfavorable from the point of view of the separation of gas mixtures containing carbon dioxide. It worsens the selectivity, facilitating the transport of mixture components being separated from carbon dioxide (e.g., methane) [17,37,38]. However, it should be noted that the sample investigated here still returned to its original state after determining each CO_2_ isotherm, both in terms of its initial weight and the sorption capacity for carbon dioxide. The phenomenon is therefore reversible in the range of pressures and temperatures at which this research was conducted. This is in line with the view presented in the available literature regarding the impact of CO_2_ on the condition of glassy membranes [37,39,40,41].

### 2.2. Solubility of CO_2_, CH_4_, O_2_ and N_2_ According to DMS Model

The sorption isotherms of carbon dioxide, methane, oxygen and nitrogen in the polyimide-based membrane sample were described using the Dual Mode Sorption (DMS) model, which is presented in detail in Section 3.4. The model assumes that the gas sorption occurs simultaneously in the matrix of the polymer and its microvoid region, called the fractional free volume (FFV). The first sorption share is later referred to as Henry sorption and the second one as Langmuir sorption. Although the DMS model is a popular tool used to describe solubility in polymer membranes, a significant problem is the determination of its coefficients, which is widely discussed in the literature [17,18,19,25,29]. It has often been reported, among others, that for one experimental sorption isotherm, it is possible to derive different sets of model coefficients with good numerical fitting accuracy using the nonlinear least-squares method [19,25,26,42,43,44]. Thus, in the current study, the fitting was performed separately for the experimental points expressed as C = f(p) and S = f(p). In the first case, hereinafter referred to as the model with minimized squared concentration differences, isotherms were approximated by Equation (4). The second case is hereinafter referred to as the model with minimized squared solubility differences, and coefficients were estimated using Equation (5). Therefore, each experimental data set concerning a single sorption isotherm is accompanied by two sets of k_D_, b and C′_H_ coefficients. These coefficients are dependent on temperature, which is shown graphically in Figures S1–S3 (points) in the Supplementary Material. As can be seen in these figures, both approximation methods result in rather similar sets of k_D_, b and C′_H_ coefficients for all gases. This temperature dependency of k_D_, b and C′_H_ was described by Equations (6)–(8), respectively. Their coefficients were also determined using the least-squares method and are summarized in Table 1.

**Figure 4:**
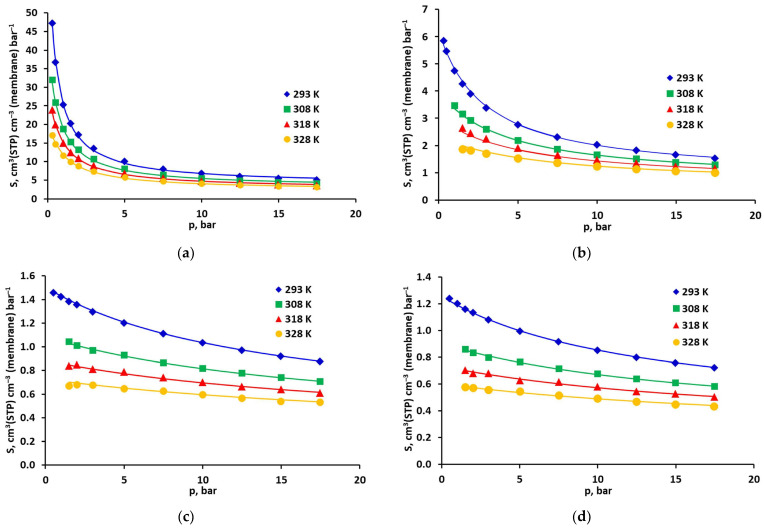
Solubility of pure (**a**) CO_2_, (**b**) CH_4_, (**c**) O_2_, and (**d**) N_2_, in the polyimide-based membrane from the UBE UMS-A2 module. Points represent experimental data, and lines represent DMS model predictions (obtained by minimizing the squared solubility differences).

Figure 4 graphically presents (as lines) sorption isotherms of the four gases, which were calculated using the DMS model and its coefficients from Table 1 for the case with minimized squared solubility differences. Experimental data (for sorption runs) are also shown in this figure as points for reference. Similarly determined isotherms for the case with minimized squared concentration differences are given in Figure S4 in the Supplementary Material. The qualitative and quantitative agreement of the experimental data with the isotherms calculated using the DMS model is good for all gases, regardless of the approximation method. It can be seen in these figures, and it is also confirmed by the low values of the mean relative error (RE), and standard error of the estimate (SEE), which are presented in Table 1. The errors are defined by Equations (13) and (14), respectively. SSE was suggested to assess the quality of the fit in [25]. While the mean relative error values are similar for both approximation methods, the SSEs are significantly lower for each gas in the case with minimized squared solubility differences. This indicates greater accuracy of fitting in the highly nonlinear region. However, it should be noted that for gases such as O_2_ or N_2_, and even methane, the solubility is low at low pressures and the number of experimental points that can be determined with sufficient accuracy is limited. Thus, an uncertainty arises especially in the determination of b and C′_H_, which describe the sorption in the Langmuir area. Therefore, the physical significance of the obtained coefficients of the DMS model was also assessed in this study based on other premises.

As can be seen in Figure S1 in the Supplementary Material, values of Henry’s solubility coefficient for individual gases are as follows: k_D_ (CO_2_) > k_D_ (CH_4_) > k_D_ (O_2_) > k_D_ (N_2_). This relationship is consistent with the widely available literature data for polymer membranes [32,33,45,46]. The heat of solubility in the Henry region, derived from –ΔH_D_/R (cf. Table 1), is the highest for CO_2_ (11.7–11.9 kJ mol^−1^) and similar for the other gases (9.7–10.1 kJ mol^−1^). As can be seen in Figure S3 in the Supplementary Material, the Langmuir affinity coefficient (b) of CO_2_ is several times higher than that of CH_4_ and two orders of magnitude higher than that of oxygen and nitrogen. The heat of adsorption in FFV, derived from −ΔH_L_/R (cf. Table 1), is slightly higher for CO_2_ (18.7–20.2 kJ mol^−1^) compared to CH_4_ (17.2–19.2 kJ mol^−1^). –ΔH_L_ of oxygen and nitrogen are lower and amount to, respectively, 12.7–14.4 kJ mol^−1^ and 9.7–14.3 kJ mol^−1^. The values of the Langmuir affinity coefficient and heat of adsorption obtained in this study are typical for the adsorption of gases in porous solids as well as in FFV of glassy polymers. According to Scholes, the absolute values of the heat of adsorption in Matrimid 5218 are 11.0 kJ mol^−1^ for CH_4_, 3.4 kJ mol^−1^ for N_2_ and 14.9 kJ mol^−1^ for CO_2_ [30]. Stevens, on the other hand, gives ΔH_s_ values at 10 atm for HAB-6FDA polyimide: 13 kJ mol^−1^ for N_2_ and 11 kJ mol^−1^ for CH_4_ and CO_2_ [31].

Table 1 also lists the values of the −C′_H1_/C′_H0_ ratio, i.e., the intersection of the C′_H_ = f(T) line with the abscissa axis. This value may be referred to the temperature at which the fractional free volume in the polymer should theoretically disappear [29]. Except for nitrogen, which dissolves least in the tested polymer, the value of this ratio is similar for individual gases in both approximation methods. Its values for “light” gases (CH_4_, O_2_ and N_2_—case with minimized squared solubility differences) are also similar, ranging from 447 to 479 K. This can be considered as additional evidence of good quality of the model fit to the experimental results, as well as the consistency of the obtained results.

**Figure 5:**
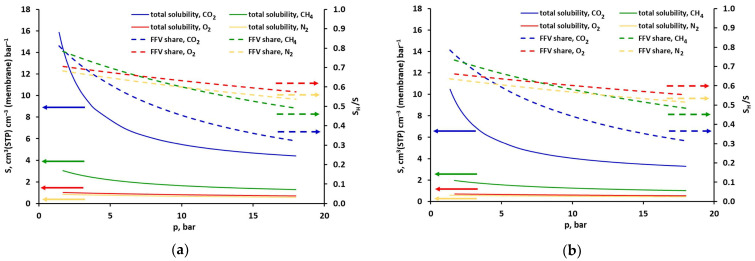
Comparison of total solubility and FFV share in total solubility of CO_2_, CH_4_, O_2_, and N_2_ in the polyimide-based membrane at (**a**) 308 K and (**b**) 328 K for minimized squared solubility differences.

The solubility of all gases in the investigated polyimide-based sample, determined from the DMS model for temperatures of 308 and 328 K, is presented in Figure 5 as a function of pressure (in the case of minimizing the squared solubility differences). As can be seen in this figure, the total solubility of carbon dioxide is the highest and strongly nonlinear. It is 3–5 times higher than that of methane. This, in turn, dissolves in the tested polyimide membrane sample much (2–3 times) better than oxygen and nitrogen. In the case of the latter three gases, adsorption in FFV has a dominant (>50%) share in the total solubility in practically the entire pressure range. As for CO_2_, above 8 bar, the share of sorption in the polymer matrix becomes dominant in the total solubility. Therefore, if only solubility is taken into account, from the point of view of separating carbon dioxide from other gases, it is most advantageous if its partial pressure in the mixture does not exceed 8 bar. In this situation, it can be expected that in the presence of this component, oxygen and nitrogen will practically not adsorb in the fractional free volume (FFV). This will limit their overall solubility in the membrane material and, consequently, their permeation through the membrane [30]. Methane is a more significant competitor to CO_2_ for access to the adsorption active centres in FFV. However, as shown in our previous work [19] in the case of simultaneous adsorption of both gases in the FFV of the polysulfone-based sample, the ratio of pure and mixed solubility of CO_2_ and CH_4_ was in the range of 0.89–0.93 and 0.59–0.69, respectively.

**Figure 6:**
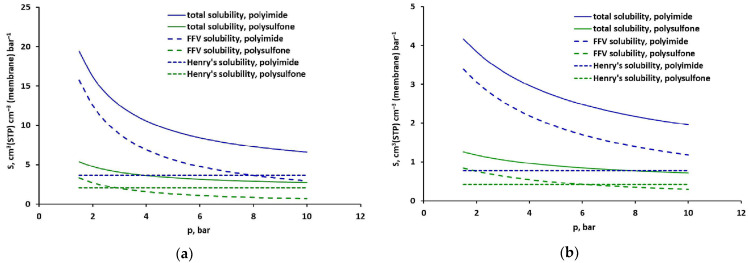
Comparison of total solubility and FFV share in total solubility of (**a**) CO_2_, and (**b**) CH_4_ in the polyimide- and polysulfone-based membrane at 293 K for minimized squared solubility differences.

The analysis concerning the solubility and permeability of CO_2_, CH_4_, O_2_ and N_2_ in the polyimide-based membrane studied here, when they occur in a mixture, will be the subject of a separate work. Whereas Figure 6 compares the solubility of pure carbon dioxide and methane in samples of polyimide- and polysulfone-based glassy polymers [19]. As can be seen in this figure, the total solubility of both gases is about 2.2–3.6 times higher in the polyimide-based sample studied in this work. The solubility of CO_2_ and CH_4_ in the polyimide-based matrix itself is also higher (about 1.8 times) than in the case of the polysulfone-based matrix. However, it is worth noting that both gases adsorb significantly better in the FFV of the polyimide-based sample over the entire pressure range. In the case of the polysulfone-based sample, solubility in the Langmuir region has a significant (>50%) share in the total solubility only at low pressure, below 3 bar for CO_2_ and 6 bar for CH_4_.

### 2.3. Permeance and Diffusivity of CO_2_, CH_4_, O_2_ and N_2_

**Figure 7:**
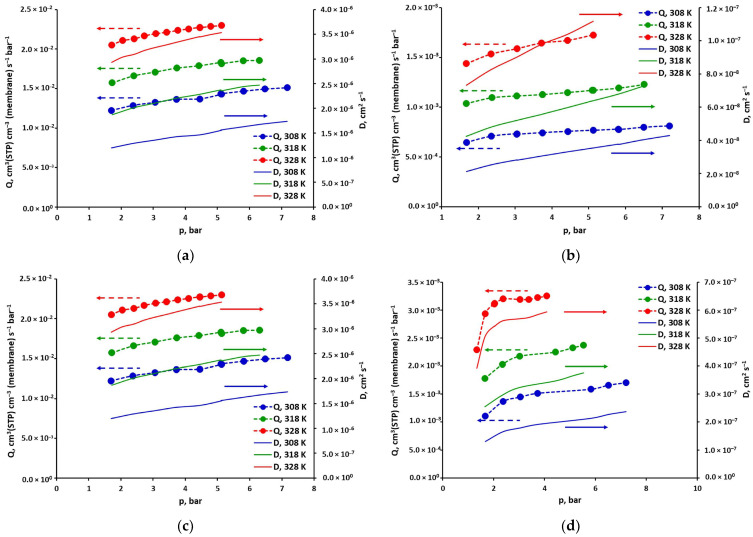
Permeance (points) and diffusivity (solid lines) of pure (**a**) CO_2_, (**b**) CH_4_, (**c**) O_2_, and (**d**) N_2_ in the polyimide-based membrane from the UBE UMS-A2 module.

The permeance coefficients of pure gases in the UBE UMS-A2 module with a polyimide-based membrane, determined experimentally according to the methodology presented in Section 3.2, are presented graphically in Figure 7. The dotted lines connecting the experimental points serve only as an eye guide. The values of these coefficients are arranged in the following order: Q (CO_2_) > Q (O_2_) > Q (N_2_) > Q (CH_4_). This trend is typical for gas permeation in polyimide-based membranes [17,46,47,48,49,50]. As can also be seen in Figure 7, the permeance coefficients increase with the increase in temperature. This tendency is pronounced more the slower the gas permeates through the membrane. Thus, the permeance coefficient at the highest temperature (328 K) is ~1.3 times greater for CO_2_, ~1.7 times for O_2_ and ~2.2 times in the case of N_2_ and CH_4_, with respect to the permeance coefficient at the lowest temperature (308 K). For any gas, permeance also increases with increasing pressure over the entire measurement pressure range. Depending on the gas and temperature, the difference between the values obtained for the highest and lowest pressure ranges from 10 to 30%. The pressure range is different for each gas, which is closely related to the permeation rate through the membrane. For a given membrane module area, the permeate flux at maximum pressure was approximately 95% of the feed flux.

The solution-diffusion model provides a simple relationship between the permeability (or permeance), solubility and diffusivity (Equation (1)). This allows one of these quantities to be determined, once two others have been directly measured, including usually the permeability/permeance [26,27,39,44,51]. The diffusion coefficients of the four gases investigated in the current study (pure CO_2_, CH_4_, O_2_ and N_2_) were determined based on their solubility and permeance in the polyimide-based membrane sample at 308, 318 and 328 K. In Figure 7, the dependence of these diffusivities on gas pressure is presented graphically as solid lines. The appropriate numerical values are given in Tables S1–S4 in the Supplementary Material. In the general case, the diffusion coefficients of pure CO_2_, CH_4_, O_2_ and N_2_ increase monotonically and almost linearly with the increase in their pressure, as can be seen in Figure 7.

These diffusion coefficients of CO_2_, CH_4_, O_2_ and N_2_ determined from the solution-diffusion model and experimental permeances and solubilities were described using the partial immobilization model, presented in Section 3.4. The linearized form of Equation (9) is shown graphically in Figure S5 in the Supplementary Material. The diffusivity in the polymer matrix D_D_ is the slope of the straight line in this approach. In turn, the parameter F can be determined from the intercept (D_D_ K F). It represents the ratio of the diffusion coefficients in the Langmuir and Henry regions. Both parameters are presented in Table 2 for all gases and for the DMS model coefficients approximated by minimizing the squared solubility differences. The coefficient of determination R^2^ in calculating the diffusion coefficients is very high in the case of CO_2_ and CH_4_ (0.993–0.999), and high for O_2_ (0.984–0.99). This is another indication of the comprehensive consistency of the experimental solubility and permeance values. The quality of fit is lowest with nitrogen. The coefficients of determination are the lowest here (0.937–0.974), although still acceptable. In this case, it is important to note that the solubility of N_2_ in the tested glassy polymer sample is very low, especially at lower pressure and higher temperature, and therefore difficult to measure with sufficient accuracy.

As can be seen in Table 2, pure CO_2_ is immobilized in FFV (F = 0) and CH_4_ is somewhat mobile in this area (F ≠ 0). The same was true for the adsorption of these gases in the FFV of the polysulfone-based sample studied in our previous work [19]. The lack of CO_2_ mobility in the FFV, with the strong affinity shown above towards the tested membrane, probably indicates that pure CO_2_ is condensing in this area. In turn, methane, which has a much lower affinity for the tested glassy polymer, most likely forms an adsorbed layer on the surface of the polymer voids and its excess fills the rest of the FFV as a gas. Mobility was also not observed in the case of oxygen and nitrogen (F = 0). This is most likely due to the fact that both of these gases adsorb much less strongly than CH_4_ in the FFV of the polyimide membrane sample tested here (cf. Figure 5). In particular, their affinity towards polymer surfaces is much lower, as illustrated by the value of the b parameter of the DMS model (cf. Figure S3 in the Supplementary Material). In such a situation, the number of active sites available for the adsorption of these gases may be excessive. It is therefore likely that each particle that enters the FFV is adsorbed on its surface and does not appear in this region in the gas phase.

**Figure 8:**
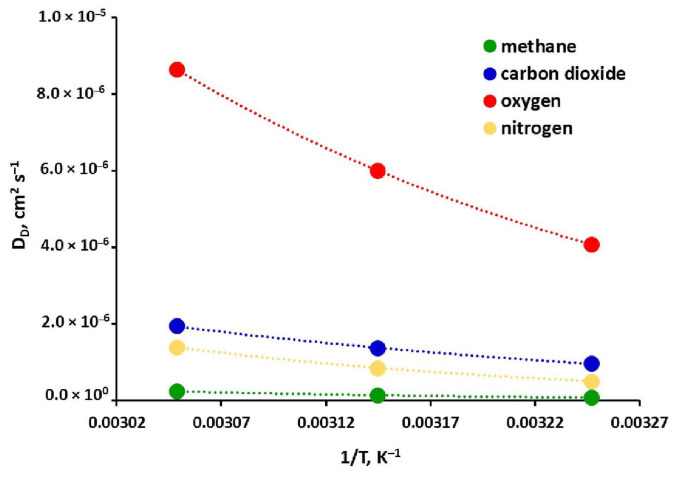
Temperature dependence of diffusion coefficient in the Henry region of the polyimide-based membrane sample from the UBE UMS-A2 module.

The diffusion coefficients in the Henry region (D_D_) derived from the partial immobilization model and presented in Table 2 are temperature dependent. This dependency is presented graphically (as points) for all gases in Figure 8 and may be described by an Arrhenius-type relationship (Equation (12)). Its coefficients were determined using the least-squares method and are summarized in Table 3. The fit is very good in this case, as evidenced by the consistency of the lines and points in Figure 8, as well as the values of the coefficient of determination (~1, cf. Table 3). This may be another indicator of the overall good consistency of both experimental data and model coefficients presented in this study. As can be seen in Figure 8, the diffusivity values in the matrix of the tested glassy polymer are arranged according to the size of the permeating molecule: D_D_ (O_2_) > D_D_ (CO_2_) > D_D_ (N_2_) > D_D_ (CH_4_). The diffusion of oxygen in this region is about 4–5 times faster than that of carbon dioxide, an order of magnitude faster than that of nitrogen, and nearly two orders of magnitude faster than that of methane. CH_4_ and N_2_ are also characterized by the highest values of diffusion activation energy, equal to 42.85 and 50.86 kJ mol^−1^, respectively (cf. Table 3). This parameter is significantly lower for CO_2_ and O_2_ (respectively, 29.66 and 31.54 kJ mol^−1^). In the case of methane, diffusion is therefore a factor that significantly limits its net transport through both the polyimide-based membrane studied in this work and other glassy polymer membranes [26,47,50].

The D_D_ coefficient is a constant parameter in the partial immobilization model. In some specific cases, when F = 0 or when the gas strongly adsorbs in the fractional free volume, the overall diffusion coefficient will theoretically tend toward D_D_. In the previous work [19], it was found that in the presence of carbon dioxide, whose adsorption is dominant, the mobility of methane disappears in the FFV of the polysulfone-based sample, and the diffusion coefficient D_D_ is practically the same for pure methane and methane mixed with CO_2_. It can therefore be assumed that in the case of permeation of mixtures containing CH_4_ and CO_2_ in the polyimide-based sample studied in this work, the overall methane diffusion coefficient will also be close to the diffusivity in the Henry region. A similar conclusion can also be drawn for oxygen and nitrogen, which adsorb much less strongly in FFV and do not compete with adsorbing CO_2_ in this area. This means that when these three gases occur in mixtures with carbon dioxide, their permeance coefficients in the glassy polymer studied here can be determined with high probability based on the models and their coefficients presented in this work.

In order to verify the conclusions in the above paragraph, a detailed analysis regarding the diffusion of CH_4_, O_2_ and N_2_ in the tested polyimide-based membrane when they are present in a mixture will be the subject of a separate work. Moreover, such an analysis will be needed in the case of CO_2_. In our previous work [19], it was found that the presence of methane induces the mobility of carbon dioxide in the fractional free volume of a polysulfone-based sample (the parameter F for CO_2_ mixed with CH_4_ became significantly greater than 0). Moreover, in the presence of methane, the diffusion coefficient (D_D_) of CO_2_ in the Henry region of the polysulfone-based sample decreases approximately twofold. These phenomena were identifiable based on experimental results on the permeation of CO_2_/CH_4_ mixtures and cannot be predicted by a partial immobilization model based on parameters obtained for pure gases.

### 2.4. Selectivity of CO_2_, CH_4_, O_2_ and N_2_

**Figure 9:**
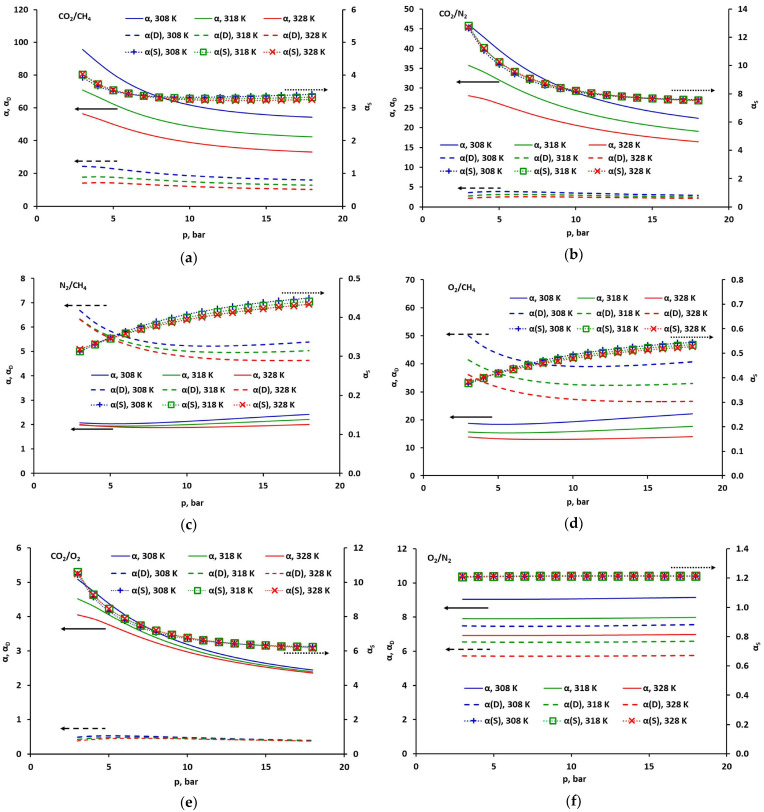
Comparison of total (solid lines), solubility (dotted lines with points) and diffusivity (dashed lines) selectivity, for (**a**) CO_2_/CH_4_, (**b**) CO_2_/N_2_, (**c**) N_2_/CH_4_, (**d**) O_2_/CH_4_, (**e**) CO_2_/O_2_, (**f**) O_2_/N_2_. The case of the DMS model with minimized squared solubility differences.

An important indicator that determines the efficiency of the separation process is the selectivity (defined by Equation (2)). In the case presented in this work, where the permeance of pure gases is investigated, the discussion will focus on ideal selectivity. Figure 9 shows the ideal total, solubility and diffusivity selectivity for various gas pairs. The solubility and diffusion coefficients necessary to calculate the appropriate selectivities were determined using the DMS and partial immobilization models and the parameters of these models are given in Table 1 and Table 2. The ideal selectivity was determined as the product of solubility and diffusivity selectivity (Equation (2)). In each case, the faster-permeating component was compared to the slower-permeating component. The numerical values of the selectivity coefficients are presented in Tables S5–S7 in the Supplementary Material.

With respect to the polyimide sample studied in this work, as well as to other glassy polymers, the four gases considered here present the entire spectrum of possible combinations of solubility-diffusion properties. Carbon dioxide dissolves best in such materials and also diffuses very well. Methane, in turn, dissolves very well, but diffuses the slowest in both the polyimide-based and polysulfone-based samples [19]. Oxygen, on the other hand, diffuses the fastest in the polyimide-based membrane studied here, but dissolves very poorly, similarly to nitrogen, whose diffusion is, however, very slow. This is reflected in the dependencies presented in Figure 9. Thus, for pairs with CO_2_ (Figure 9a,b,e), the ideal selectivity decreases monotonically with increasing pressure. Initially, this decrease is due to a simultaneous decrease in solubility selectivity, and after it stabilizes, a further decrease in the ideal total selectivity is caused by a decrease in diffusivity selectivity, which at higher pressures decreases monotonically with increasing pressure for each gas pair. For CO_2_/O_2_ and CO_2_/N_2_ pairs, solubility selectivity is the dominant factor. It is in the range of 6.2–10.5 and 7.5–12.7, respectively. In the case of the CO_2_/O_2_ pair, the diffusivity selectivity is less than 1; therefore, the resultant ideal selectivity is relatively low, in the range of 2.3–5.1. In combination with nitrogen, carbon dioxide also dominates in terms of diffusion, and although the diffusivity selectivity is relatively low (2.2–2.8), the overall ideal selectivity is high (16.4–46), especially at lower temperatures and pressures. In the case of the CO_2_/CH_4_ pair, the solubility selectivity is low (3.25–4), and the dominant factor is the diffusivity selectivity, in the range of 10.2–24.4. This translates into the highest ideal selectivity of all gas pairs (33–95.7), which is strongly dependent on temperature and pressure. However, it should be taken into account that methane negatively affects both the solubility and the diffusion rate of CO_2_ in glassy polymers, which results in a reduction in the CO_2_/CH_4_ selectivity in the actual gas mixture [19,25,26,27,28].

The overall ideal selectivity of O_2_/CH_4_ (cf. Figure 9c) and N_2_/CH_4_ (cf. Figure 9d) initially decreases slightly, and after exceeding the pressure of approx. 8 bar, it also increases slightly with pressure. At a temperature of 308 K, for example, it varies in the ranges of 18.4–22.2 and 1.9–2.4, respectively. In this case, the increase in solubility selectivity with pressure is compensated by a simultaneous decrease in diffusivity selectivity. With respect to oxygen and nitrogen, methane is the component that dissolves better in the tested polyimide-based sample; however, the solubility selectivity of O_2_/CH_4_ and N_2_/CH_4_ is relatively low, in the range of 0.38–0.54 and 0.31–0.45, respectively. The dominant factor in the case of these pairs is therefore the diffusivity selectivity, very high for the O_2_/CH_4_ pair (26.6–50.1) and moderate for the N_2_/CH_4_ pair (4.6–6.7).

In the case of oxygen and nitrogen, whose solubility is similar, the total selectivity will obviously be determined by the diffusivity selectivity, which is in the range of 5.7–7.6. In this case, where the solubility, and, in particular, the adsorption in the fractional free volume, is very low, both the total selectivity and its components are practically constant over the entire pressure range.

## 3. Materials, Methods and Model

### 3.1. Membrane Sample and Gases

The sample of a polyimide-based membrane for sorption experiments was derived from the hollow-fiber UMS-A2 module (UBE Corporation, Ube, Japan). The fibers were cut into pieces of about 2 mm in length, forming a sample with an initial weight of 124.95 mg.

In the sorption experiments carbon dioxide of at least 99.995% purity supplied by Messer (Messer Polska Sp. z o.o., Chorzów, Poland), methane of at least 99.95% purity supplied by Siad (Siad Poland Sp. z o.o., Ruda Śląska, Poland), oxygen of at least 99.9995% purity supplied by Messer, nitrogen of at least 99.9999% purity supplied by Siad and helium of at least 99.9999% purity supplied by Air Liquide (Air Liquide Polska Sp. z o.o., Dąbrowa Górnicza, Poland) were used.

In permeation experiments, the UMS-A2 module was used. Its maximum working temperature and pressure are 328 K and 15 bar, respectively. Technical-grade gases were used in these studies: carbon dioxide from Siad, methane from Air Liquide, and nitrogen and oxygen from Messer.

### 3.2. Permeation Experiments

**Figure 10:**
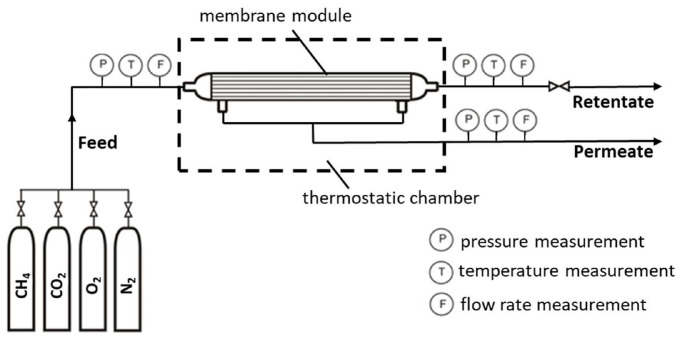
The experimental installation for investigating gas permeation.

The investigation of permeation of pure gases (CH_4_, N_2_, O_2_ and CO_2_) was carried out in the experimental installation, the diagram of which is shown in Figure 10.

The main element of the installation is the tested membrane module, which in this case was the UMS-A2 module with a bundle of densely packed thin hollow fibers made of modified polyimide. Clean gas was taken from one of the pressure cylinders and introduced into the membrane module through a control valve. There, it flowed into the hollow fibers, partially dissolved in the membrane material, and then diffused into the interfiber space. From there, it was discharged to the outside of the module as permeate. The remaining part of the inlet gas that did not pass through to the permeate side of the membrane was collected at the outlet of the module as retentate. The flow rate, pressure and temperature of the inlet gas, retentate and permeate were measured and controlled during the tests.

The tests were carried out at temperatures of 35, 45 and 55 °C. The set temperature was maintained by placing the membrane module in a thermostated chamber with forced air circulation (Memmert GmbH, Schwabach, Germany), which ensured the stability of the temperature with an accuracy of ±0.5 °C. The feed pressure was varied in the range from 1.3 to a maximum of 7.2 bar (depending on the type of gas and the set temperature) at a given feed gas flow rate and constant temperature. The inlet gas flow rate was selected so that for each gas the ratio of permeate flow rate to inlet gas flow rate was in the range of 10–95%. Therefore, the feed gas flow rate was 38 cm^3^ min^−1^ for CH_4_, 50 cm^3^ min^−1^ for N_2_, 500 cm^3^ min^−1^ for O_2_ and 800 cm^3^ min^−1^ for CO_2_. Free gas flow was ensured on the permeate side. In order to measure the permeate flow rate, flowmeters with different ranges were used, depending on the gas and its pressure. The flowmeters were selected in such a way as to ensure that the measuring range of a given device was optimally matched to the actual permeate flow. All flowmeters used are calibrated for nitrogen, and in the case of measuring other gases, a calibration conversion factor provided by the flowmeter manufacturer was used. This conversion factor takes into account both the density of the gas and its specific heat. The following flowmeters were used to measure the permeate flow rate: 0–50 cm^3^ min^−1^ for CH_4_, 0–20 and 0–50 cm^3^ min^−1^ for N_2_, 0–500 cm^3^ min^−1^ for O_2_, and 0–500 and 0–1000 cm^3^ min^−1^ for CO_2_.

The flow rate was measured using Aalborg Mass Flow Meter (Aalborg Instruments, Aalborg, Denmark): DFM27 (0–20, 0–50, 0–200 and 0–500 cm^3^ min^−1^) and GFM17 (0–200, 0–500, 0–1000 and 0–5000 cm^3^ min^−1^), with an accuracy of ±1% of the measuring range. Pressure was measured using pressure transducers (Cole-Parmer P series, Cole-Parmer Instrument, Vernon Hills, IL, USA) with an accuracy of 0.1 psi. The temperature was measured using a Cole-Parmer transducer, which provided an accuracy of ±0.1 °C. Each day after testing, the membrane installation was flushed with pure nitrogen. The purging was carried out for approximately 15 min, passing approximately 100 cm^3^ min^−1^ of nitrogen at a pressure of 4 bar.

### 3.3. Sorption Experiments

A microbalance (IGA003, Hiden Isochema Ltd., Warrington, UK) was used for gravimetric measurement of CO_2_, CH_4_, O_2_ and N_2_ sorption isotherms. It has a weighing resolution of 0.2 μg, the buoyancy force correction and is fully thermostated to eliminate the effect of ambient temperature. The temperature variations in the system do not exceed ±0.2 K. The IGA microbalance is described in detail in [52]. The measurements were done in the static mode. This means that the reactor with the sample container was filled with a gas until a desired pressure was reached. Then, the gas supply was cut off. In this state, changes in sample mass were automatically recorded and monitored until the equilibrium was established.

After loading into the IGA reactor, the sample was degassed under vacuum for 24 h. Afterwards, its density was measured in helium. Therefore, the pressure in the reactor was changed in steps of 500 mbar over a range of 2–18 bar. The temperature was kept constant at 293 K. Thus determined density was equal to 1.360 g cm^−3^. The sample was also fully degassed before each temperature and gas change.

The isotherms of CO_2_, CH_4_, O_2_ and N_2_ sorption were measured at a given temperature of 293, 308, 318 or 328 K in the pressure range of 0–17.5 bar. Each isotherm was determined in two runs: the sorption (when the pressure was increased) and desorption (when the pressure was decreased). The pressure in the IGA chamber was changed with a given step and a rate of 200 mbar min^−1^. In the case of a single isotherm point, the measurement ended if one of two criteria was met: the measured change in the sample mass reached 99.8% of the predicted asymptotic value or the measurement time for a given point exceeded 30 min.

### 3.4. Dual Mode Sorption Model

The Dual Mode Sorption (DMS) model was assumed [17,18,25,29,53] in order to theoretically describe the sorption isotherms. Two fractions: C_Di_ and C_Hi_ are considered in this model with respect to the concentration of the i-th gas in a glassy polymer. The first one is described by Henry’s law and concerns a gas dissolved in the polymer matrix. The second one is described by the Langmuir model. It refers to the gas fraction adsorbed in the fractional free volume (FFV) of the glassy polymer. The sum of both shares can be given as:

(4) $$C_{i}=C_{Di}+C_{Hi}=k_{Di}p_{i}+C_{Hi}^{\prime }\frac{b_{i}p_{i}}{1+b_{i}p_{i}}$$

where k_D_ is Henry’s solubility coefficient (cm^3^ (STP) cm^−3^ bar^−1^), b is the Langmuir affinity coefficient (bar^−1^) and C′_H_ is the Langmuir saturation constant (cm^3^ (STP) cm^−3^). Since in this investigation the assumption of ideal gas behavior is valid over the entire range of pressures and temperatures, partial pressure is used instead of fugacity (i.e., the effective pressure of an imperfect gas) [24,43,54]. Moreover, as reported in the literature [55,56] the estimations which use pressures and fugacities have often the same accuracy and lead to similar results.

A simple transformation of Equation (4) leads to the following expression describing the solubility S (cm^3^ (STP) cm^−3^ bar^−1^):

(5) $$S_{i}=k_{Di}+C_{Hi}^{\prime }\frac{b_{i}}{1+b_{i}p_{i}}$$

The parameters of the DMS model depend on temperature. The temperature dependence of k_Di_ and b_i_ can be given as an Arrhenius type equation [17,25,57]:

(6) $$k_{Di}=k_{D0i}exp\left(\frac{-∆H_{Di}}{RT}\right)$$

(7) $$b_{i}=b_{0i}exp\left(\frac{-∆H_{Li}}{RT}\right)$$

In the above equations k_D0i_ and b_0i_ are pre-exponential coefficients in cm^3^ (STP) cm^−3^ bar^−1^ and bar^−1^, respectively, ΔH_Di_ is the heat of sorption in the Henry’s region and ΔH_Li_ is the heat of adsorption in FFV in kJ mol^−1^, T is the temperature in K and R is the gas constant in kJ mol^−1^ K^−1^. According to [29], the Langmuir saturation constant C′_H_ can be related to the specific volume of FFV in the polymer, which varies linearly with temperature. Therefore, the linear relationship between C′_H_ and T can be expressed as:

(8) $$C_{Hi}^{\prime }=C_{H0i}^{\prime }T+C_{H1i}^{\prime }$$

where C′_H0i_ is in cm^3^ (STP) cm^−3^ K^−1^ and C′_H1i_ is in cm^3^ (STP) cm^−3^. The value of C′_H_ decreases with an increase in temperature until it reaches 0 at T = −C′_H1i_/C′_H0i_. In [29] this was interpreted as a transition of the polymer from a glassy to a rubbery state.

Assuming the validity of Fick’s law in the solution-diffusion and DMS models, the diffusion coefficient may be derived in the following form [29] for pure gases:

(9) $$D_{i}=D_{Di}\left[\frac{1+\frac{F_{i}\cdot K_{i}}{{\left(1+b_{i}p_{i}\right)}^{2}}}{1+\frac{K_{i}}{{\left(1+b_{i}p_{i}\right)}^{2}}}\right]=D_{Di}\left[\frac{{\left(1+b_{i}p_{i}\right)}^{2}+F_{i}\cdot K_{i}}{{\left(1+b_{i}p_{i}\right)}^{2}+K_{i}}\right]$$

where K is a dimensionless parameter, temperature-dependent, which describes a relation between sorption in the Henry’s and Langmuir regions:

(10) $$K_{i}=\frac{C_{Hi}^{\prime }\cdot b_{i}}{k_{Di}}$$

In turn, dimensionless constant F is the ratio of the diffusion coefficient in the Henry region (D_D_) and the diffusion coefficient in the Langmuir region (D_H_).

(11) $$F_{i}=\frac{D_{Hi}}{D_{Di}}$$

The temperature dependence of D_Di_ may be given as Arrhenius type relationship:

(12) $$D_{Di}=D_{D0i}exp\left(\frac{-E_{Di}}{RT}\right)$$

where D_D0i_ is a pre-exponential coefficient in cm^2^ s^−1^, and E_Di_ is the activation energy of diffusion in kJ mol^−1^. The set of Equations (9)–(12) is referred in the literature [29] and also in this work as the partial immobilization model.

Nonlinear regression was used to determine the DMS model parameters for each gas and each temperature independently, based on experimental isotherms. The squared concentration differences were minimized using Equation (4) and the squared solubility differences were minimized using Equation (5). For each gas, the resulting set of k_Di_, b_i_ and C′_Hi_ was regressed over the experimental temperature range. Two indicators were used to assess the quality of fit, i.e., the mean relative error (RE) and the standard error of estimate (SEE) [25]. They can be expressed as follows:

(13) $$RE=\frac{\sum_{j}\frac{\left|y_{j,exp}-y_{j,calc}\right|}{y_{j,exp}}}{n}$$

(14) $$SEE=\sqrt{\frac{\sum_{j}{\left(y_{j,exp}-y_{j,calc}\right)}^{2}}{n-p}}$$

where y_j,exp_ is an experimental point j and y_j,calc_ is the value of the corresponding point calculated with the model, n is the number of experimental points used in the regression, and p is the number of model parameters.

## 4. Conclusions

The factors influencing the mass transport of the four main biogas components in a glassy polyimide-based membrane from the UBE UMS-A2 module are analyzed in this work. The module is used in a developed hybrid adsorption–membrane process for CO_2_ removal from multicomponent gas mixtures and especially for biogas separation. It was found that the concentrations of pure CO_2_, CH_4_, O_2_ and N_2_ in a sample of this membrane, determined gravimetrically in the temperature and pressure range of 293–328 K and 0–18 bar, respectively, are consistent with those of other polyimide-based membranes. In the case of carbon dioxide, the effect of membrane swelling was observed at temperatures of 293 and 303 K and for pressures higher than 3 bar. These experimentally measured isotherms of CO_2_, CH_4_, O_2_ and N_2_ sorption were used to calculate the coefficients of the Dual Mode Sorption model. This model allows theoretical prediction of the solubility of these gases in pure and mixed states. The estimated model coefficients were examined for their consistency across different fitting methods of experimental data and the model, good fitting accuracy and comparison with existing literature data, in order to verify their physical plausibility. One of the conclusions was that the adsorption of methane, oxygen and nitrogen in the fractional free volume (FFV) has a dominant share (>50%) in their total solubility over practically the entire pressure range. As for CO_2_, above 8 bar, the share of sorption in the polymer matrix becomes dominant in the total solubility.

The permeances of the four main biogas components in the glassy polyimide-based membrane were determined at a temperature range of 308–328 K and it was found that they increase with increasing pressure over the entire measurement pressure range. Based on these data and on the solubility of pure CO_2_, CH_4_, O_2_ and N_2_ calculated at 308, 318 and 328 K from the DMS model, the appropriate diffusion coefficients in the sample of the tested polyimide-based membrane were determined using the solution-diffusion model. These diffusivities were described by the partial immobilization model. It was found that the diffusion of oxygen in the investigated sample is the fastest and about 4–5 times faster than CO_2_. This makes it competitive with CO_2_ in terms of permeation through the membrane, despite its low solubility. As a result, the ideal CO_2_/O_2_ selectivity is relatively low and lies in the range of 2.3–5.1. On the other hand, methane, which is competitive in solubility even with CO_2_, was found to diffuse the slowest in the polyimide-based sample material. As a result, it is also the slowest permeating gas in a membrane made of this polymer. This translates into the very high CO_2_/CH_4_ ideal selectivity (33–95.7), which is, however, strongly dependent on temperature and pressure. In the case of the CH_4_ and N_2_ pair, the significant advantage of the former in terms of solubility is compensated in an almost equivalent way by the advantage of the latter in terms of diffusion rate. The resulting ideal N_2_/CH_4_ partition coefficient is therefore very low (1.9–2.4), and the two gases are difficult to separate from each other.

A methodology developed to predict the actual transport coefficients of mixture components in a glassy membrane material was discussed, taking as examples four gases representing the entire spectrum of possible combinations of solubility and diffusion properties. Taking into account the known limitations of DMS and partial immobilization models, their coefficients concerning CH_4_, O_2_ and N_2_ or CO_2_, O_2_ and N_2_ can be used to estimate the permeance of these gases in their mixtures. However, a detailed analysis regarding the diffusion of all four gases in the tested polyimide-based membrane is necessary when they are present in a mixture. This is especially true when CO_2_ is accompanied by methane and such an analysis will be the subject of a separate work.

## Figures and Tables

**Table 1:** DMS model coefficients in the case of sorption of CO_2_, CH_4_, O_2_ and N_2_ in the polyimide-based membrane from the UBE UMS-A2 module.

	DMS Model with Minimized Squared Concentration Differences			DMS Model with Minimized Squared Solubility Differences	
Gas	CO_2_	CH_4_		CO_2_	CH_4_
k_D0_ ^1^	2.295 × 10^−2^	1.029 × 10^−2^		2.914 × 10^−2^	1.479 × 10^−2^
−ΔH_D_/R ^2^	1405.0	1219.8		1426.0	1169.0
C′_H0_ ^3^	−0.395	−0.110		−0.334	−0.101
C′_H1_ ^4^	157.6	51.02		129.3	45.32
b_0_ ^5^	5.798 × 10^−4^	2.304 × 10^−4^		5.954 × 10^−4^	1.302 × 10^−4^
−ΔH_L_/R ^2^	2253.0	2072.0		2426.0	2311.0
−C′_H1_/C′_H0_ ^6^	399.0	462.1		386.9	446.9
RE ^7^	2.82	2.30		3.04	2.03
SEE ^8^	5.11 × 10^−1^	3.73 × 10^−2^		1.17 × 10^−1^	3.94 × 10^−3^
Gas	**O_2_**	**N_2_**		**O_2_**	**N_2_**
k_D0_ ^1^	5.721 × 10^−3^	5.768 × 10^−3^		6.584 × 10^−3^	5.768 × 10^−3^
−ΔH_D_/R ^2^	1187.2	1183.1		1176.0	1183.1
C′_H0_ ^3^	−0.112	−0.108		−0.088	−0.074
C′_H1_ ^4^	50.75	43.72		42.08	33.83
b_0_ ^5^	3.538 × 10^−4^	1.380 × 10^−3^		1.859 × 10^−4^	2.193 × 10^−4^
−ΔH_L_/R ^2^	1526.0	1172.0		1737.0	1715.0
−C′_H1_/C′_H0_ ^6^	454.3	406.7		478.7	457.4
RE ^7^	0.83	1.13		0.82	0.75
SEE ^8^	1.54 × 10^−3^	1.36 × 10^−3^		0.8 × 10^−4^	0.41 × 10^−4^

^1^ k_D0_ is in cm^3^ (STP) cm^−3^ (membrane) bar^−1^. ^2^ −ΔH_D_/R and −ΔH_L_/R is in K. ^3^ C′_H0_ is in cm^3^ (STP) cm^−3^ (membrane) K^−1^. ^4^ C′_H1_ is in cm^3^ (STP) cm^−3^ (membrane). ^5^ b_0_ is in bar^−1^. ^6^ −C′_H1_/C′_H0_ is in K. ^7^ RE (average relative error) is in %. ^8^ SEE is in cm^3^ (STP) cm^−3^ (membrane) and in cm^3^ (STP) cm^−3^ (membrane) bar^−1^ for the squared concentration differences and squared solubility differences, respectively.

**Table 2:** Coefficients of the partial immobilization model in the case of sorption and diffusion of CO_2_, CH_4_, O_2_ and N_2_ in the polyimide-based membrane from the UBE UMS-A2 module.

	CO_2_				CH_4_		
	308 K	318 K	328 K		308 K	318 K	328 K
K	13.849	10.920	8.481		5.056	4.176	3.452
D_D_^1^	9.57 × 10^−7^	1.36 × 10^−6^	1.94 × 10^−6^		6.94 × 10^−8^	1.26 × 10^−7^	2.33 × 10^−7^
F	0	0	0		0.049	0.061	0.009
R^2^	0.993	0.996	0.996		0.999	0.999	0.999
	**O_2_**				**N_2_**		
	**308 K**	**318 K**	**328 K**		**308 K**	**318 K**	**328 K**
K	2.618	2.328	2.069		2.362	2.087	1.841
D_D_	4.08 × 10^−6^	6.00 × 10^−6^	8.65 × 10^−6^		5.01 × 10^−7^	8.43 × 10^−7^	1.39 × 10^−6^
F	0	0	0		0	0	0
R^2^	0.986	0.990	0.984		0.974	0.953	0.937

^1^ D_D_ is in cm^2^ s^−1^.

**Table 3:** Coefficients of Equation (12) to determine the temperature dependence of the diffusion coefficient in the Henry’s law region for CO_2_, CH_4_, O_2_ and N_2_ in the polyimide-based membrane from the UBE UMS-A2 module.

	CO_2_	CH_4_	O_2_	N_2_
D_D0_^1^	0.102	29.0	0.908	9.25
E_D_^2^	29.66	50.86	31.54	42.85
R^2^	0.9996	0.9994	1	1

^1^ D_D0_ is in cm^2^ s^−1^. ^2^ E_D_ is in kJ mol^−1^.

## Data Availability

Data are contained within the article and Supplementary Materials.

## Supplementary Materials

The following supporting information can be downloaded at: https://www.mdpi.com/article/10.3390/molecules30234524/s1, Table S1. Permeance, solubility and diffusivity of CO_2_ in the polyimide-based membrane from UBE UMS-A2 module; Table S2. Permeance, solubility and diffusivity of CH_4_ in the polyimide-based membrane from UBE UMS-A2 module; Table S3. Permeance, solubility and diffusivity of O_2_ in the polyimide-based membrane from UBE UMS-A2 module; Table S4. Permeance, solubility and diffusivity of N_2_ in the polyimide-based membrane from UBE UMS-A2 module; Table S5. Summary of the solubility, diffusivity and selectivity of pure CO_2_, CH_4_, O_2_ and N_2_ at 308 K in the polyimide-based membrane from UBE UMS-A2 module; Table S6. Summary of the solubility, diffusivity and selectivity of pure CO_2_, CH_4_, O_2_ and N_2_ at 318 K in the polyimide-based membrane from UBE UMS-A2 module; Table S7. Summary of the solubility, diffusivity and selectivity of pure CO_2_, CH_4_, O_2_ and N_2_ at 328 K in the polyimide-based membrane from UBE UMS-A2 module; Figure S1. Temperature dependence of the Henry’s constant in the Dual Mode Sorption (DMS) model for (a) CO_2_, (b) CH_4_, (c) O_2_ and (d) N_2_. The dotted lines are obtained from the fit; Figure S2. Temperature dependence of the Langmuir adsorption capacity in the Dual Mode Sorption (DMS) model for (a) CO_2_, (b) CH_4_, (c) O_2_ and (d) N_2_. The dotted lines are obtained from the fit; Figure S3. Temperature dependence of the Langmuir affinity constant in the Dual Mode Sorption (DMS) model for (a) CO_2_, (b) CH_4_, (c) O_2_ and (d) N_2_. The dotted lines are obtained from the fit; Figure S4. Concentration of pure (a) CO_2_, (b) CH_4_, (c) O_2_ and (d) N_2_ in the polyimide-based membrane from UBE UMS-A2 module. Points represent experimental data and lines are Dual Mode Sorption (DMS) model predictions (for minimized the squared concentration differences); Figure S5. Diffusivity of pure (a) CO_2_, (b) CH_4_, (c) O_2_ and (d) N_2_ in the polyimide-based membrane from UBE UMS-A2 module according to the linearized partial immobilization model. A straight dotted line is from the fit. The case of the DMS model with minimized the squared solubility differences.
