# Supplementary material for: Transport of Carbon Dioxide, Methane, Oxygen and Nitrogen in a Glassy Polyimide Membrane
Source: Molecules. 2025 Nov 23;30(23):4524. doi: 10.3390/molecules30234524 (PMC12693044; doi:10.3390/molecules30234524)
Supplement: Supplementary file 1 [file molecules-30-04524-s001.zip › molecules-3987756-supplementary.pdf]

Supplementary Material (ESI)

Transport of carbon dioxide, methane, oxygen and nitrogen in a glassy polyimide membrane.

Marek Tańczyk [1], *, Aleksandra Janusz-Cygan [1], Anna Pawlaczyk-Kurek [1], Łukasz Hamryszak [1] and Jolanta Jaschik [1]

[1] Institute of Chemical Engineering, Polish Academy of Sciences, ul. Bałtycka 5, 44-100 Gliwice, Poland; ajcygan@iich.gliwice.pl (A.J.-C.); ania.pawlaczyk@iich.gliwice.pl (A.P.-K.); lukasz.hamryszak@iich.gliwice.pl (Ł.H.); jjaschik@iich.gliwice.pl (J.J.)

* Correspondence: mtanczyk@iich.gliwice.pl (M.T.); Tel.: +48 32 234 69 15

THE TABLE OF CONTENTS

Table S1. Permeance, solubility and diffusivity of CO_2 in the polyimide-based membrane from UBE UMS-A2 module........ 2

Table S2. Permeance, solubility and diffusivity of CH_4 in the polyimide-based membrane from UBE UMS-A2 module........ 3

Table S3. Permeance, solubility and diffusivity of O_2 in the polyimide-based membrane from UBE UMS-A2 module........ 4

Table S4. Permeance, solubility and diffusivity of N_2 in the polyimide-based membrane from UBE UMS-A2 module........ 5

Table S5. Summary of the solubility, diffusivity and selectivity of pure CO_2, CH_4, O_2 and N_2 at 308 K in the polyimide-based membrane from UBE UMS-A2 module........ 6

Table S6. Summary of the solubility, diffusivity and selectivity of pure CO_2, CH_4, O_2 and N_2 at 318 K in the polyimide-based membrane from UBE UMS-A2 module........ 7

Table S7. Summary of the solubility, diffusivity and selectivity of pure CO_2, CH_4, O_2 and N_2 at 328 K in the polyimide-based membrane from UBE UMS-A2 module........ 8

Fig. S1. Temperature dependence of the Henry's constant in the Dual Mode Sorption (DMS) model for (**a**) CO_2, (**b**) CH_4, (**c**) O_2 and (**d**) N_2. The dotted lines are obtained from the fit........ 9

Fig. S2. Temperature dependence of the Langmuir adsorption capacity in the Dual Mode Sorption (DMS) model for (**a**) CO_2, (**b**) CH_4, (**c**) O_2 and (**d**) N_2. The dotted lines are obtained from the fit........ 10

Fig. S3. Temperature dependence of the Langmuir affinity constant in the Dual Mode Sorption (DMS) model for (**a**) CO_2, (**b**) CH_4, (**c**) O_2 and (**d**) N_2. The dotted lines are obtained from the fit........ 11

Fig. S4. Concentration of pure (**a**) CO_2, (**b**) CH_4, (**c**) O_2 and (**d**) N_2 in the polyimide-based membrane from UBE UMS-A2 module. Points represent experimental data and lines Dual Mode Sorption (DMS) model predictions (for minimized the squared concentration differences)........ 12

Fig. S5. Diffusivity of pure (**a**) CO_2, (**b**) CH_4, (**c**) O_2 and (**d**) N_2 in the polyimide-based membrane from UBE UMS-A2 module according to the linearized partial immobilization model. A straight dotted line is from the fit. The case of the DMS model with minimized the squared solubility differences........ 13

Table S1. Permeance, solubility and diffusivity of CO_2 in the polyimide-based membrane from UBE UMS-A2 module.

**308 K**				**318 K**				**328 K**			
**p**[1]	**Q**[2]	**S**[3]	**D**[4]	**p**[1]	**Q**[2]	**S**[3]	**D**[4]	**p**[1]	**Q**[2]	**S**[3]	**D**[4]
1.41	$5.81·10^{-2}$	15.86	$3.66·10^{-7}$	1.40	$6.43·10^{-2}$	12.99	$4.95·10^{-7}$	1.37	$6.98·10^{-2}$	10.47	$6.67·10^{-7}$
1.56	$6.21·10^{-2}$	15.00	$4.14·10^{-7}$	1.55	$6.96·10^{-2}$	12.32	$5.65·10^{-7}$	1.52	$7.75·10^{-2}$	9.98	$7.76·10^{-7}$
1.68	$6.44·10^{-2}$	14.36	$4.48·10^{-7}$	1.69	$7.26·10^{-2}$	11.77	$6.17·10^{-7}$	1.68	$8.11·10^{-2}$	9.53	$8.52·10^{-7}$
1.86	$6.60·10^{-2}$	13.53	$4.88·10^{-7}$	1.86	$7.38·10^{-2}$	11.17	$6.61·10^{-7}$	1.85	$8.32·10^{-2}$	9.09	$9.16·10^{-7}$
2.02	$6.58·10^{-2}$	12.92	$5.09·10^{-7}$	1.84	$7.39·10^{-2}$	11.24	$6.58·10^{-7}$	2.00	$8.45·10^{-2}$	8.74	$9.67·10^{-7}$
2.18	$6.73·10^{-2}$	12.35	$5.45·10^{-7}$	1.99	$7.54·10^{-2}$	10.78	$6.99·10^{-7}$	2.17	$8.63·10^{-2}$	8.40	$1.03·10^{-6}$
2.28	$6.82·10^{-2}$	12.02	$5.68·10^{-7}$	2.14	$7.60·10^{-2}$	10.37	$7.33·10^{-7}$	2.30	$8.77·10^{-2}$	8.16	$1.07·10^{-6}$
2.49	$6.85·10^{-2}$	11.43	$5.99·10^{-7}$	2.30	$7.80·10^{-2}$	9.98	$7.82·10^{-7}$	2.33	$8.76·10^{-2}$	8.11	$1.08·10^{-6}$
2.61	$6.95·10^{-2}$	11.10	$6.26·10^{-7}$	2.50	$7.87·10^{-2}$	9.53	$8.26·10^{-7}$	2.49	$8.93·10^{-2}$	7.85	$1.14·10^{-6}$
2.84	$7.09·10^{-2}$	10.57	$6.71·10^{-7}$	2.55	$7.91·10^{-2}$	9.41	$8.40·10^{-7}$	2.65	$8.97·10^{-2}$	7.60	$1.18·10^{-6}$
3.05	$7.06·10^{-2}$	10.13	$6.97·10^{-7}$	2.63	$7.96·10^{-2}$	9.26	$8.59·10^{-7}$				

[1] p is in bar
[2] Q is in cm^3(STP) cm^{-2} s^{-1} bar^{-1}
[3] S is in cm^3(STP) cm^{-3}(membrane) bar^{-1}, calculated from the Dual Mode Sorption (DMS) model with minimized the squared solubility differences
[4] D is in cm^2 s^{-1}, calculated from the partial immobilization model, assuming the membrane active layer thickness of 1 μm and using the appropriate permeance and solubility from this table

Table S2. Permeance, solubility and diffusivity of CH_4 in the polyimide-based membrane from UBE UMS-A2 module.

308 K				318 K				328 K			
**p**[1]	**Q**[2]	**S**[3]	**D**[4]	**p**[1]	**Q**[2]	**S**[3]	**D**[4]	**p**[1]	**Q**[2]	**S**[3]	**D**[4]
1.68	$6.46\cdot10^{-4}$	3.04	$2.12\cdot10^{-8}$	1.67	$1.04\cdot10^{-3}$	2.44	$4.24\cdot10^{-8}$	1.67	$1.44\cdot10^{-3}$	1.96	$7.32\cdot10^{-8}$
2.36	$7.08\cdot10^{-4}$	2.80	$2.53\cdot10^{-8}$	2.37	$1.10\cdot10^{-3}$	2.28	$4.82\cdot10^{-8}$	2.34	$1.54\cdot10^{-3}$	1.86	$8.27\cdot10^{-8}$
3.05	$7.33\cdot10^{-4}$	2.59	$2.83\cdot10^{-8}$	3.04	$1.11\cdot10^{-3}$	2.14	$5.21\cdot10^{-8}$	3.05	$1.59\cdot10^{-3}$	1.76	$9.02\cdot10^{-8}$
3.05	$7.28\cdot10^{-4}$	2.59	$2.81\cdot10^{-8}$	3.74	$1.13\cdot10^{-3}$	2.02	$5.58\cdot10^{-8}$	3.72	$1.65\cdot10^{-3}$	1.68	$9.79\cdot10^{-8}$
3.74	$7.44\cdot10^{-4}$	2.42	$3.07\cdot10^{-8}$	3.75	$1.13\cdot10^{-3}$	2.02	$5.59\cdot10^{-8}$	4.42	$1.67\cdot10^{-3}$	1.61	$1.04\cdot10^{-7}$
4.43	$7.57\cdot10^{-4}$	2.28	$3.31\cdot10^{-8}$	4.43	$1.15\cdot10^{-3}$	1.92	$5.98\cdot10^{-8}$	5.12	$1.73\cdot10^{-3}$	1.54	$1.12\cdot10^{-7}$
5.12	$7.67\cdot10^{-4}$	2.16	$3.54\cdot10^{-8}$	5.10	$1.17\cdot10^{-3}$	1.83	$6.38\cdot10^{-8}$				
5.80	$7.78\cdot10^{-4}$	2.06	$3.77\cdot10^{-8}$	5.13	$1.17\cdot10^{-3}$	1.83	$6.40\cdot10^{-8}$				
5.81	$7.74\cdot10^{-4}$	2.06	$3.76\cdot10^{-8}$	5.81	$1.19\cdot10^{-3}$	1.75	$6.80\cdot10^{-8}$				
6.50	$7.98\cdot10^{-4}$	1.97	$4.05\cdot10^{-8}$	6.51	$1.23\cdot10^{-3}$	1.69	$7.29\cdot10^{-8}$				
7.20	$8.12\cdot10^{-4}$	1.89	$4.29\cdot10^{-8}$								

[1] p is in bar

[2] Q is in cm^3(STP) cm^{-2} s^{-1} bar^{-1}

[3] S is in cm^3(STP) cm^{-3}(membrane) bar^{-1}, calculated from the Dual Mode Sorption (DMS) model with minimized the squared solubility differences

[4] D is in cm^2 s^{-1}, calculated from the partial immobilization model, assuming the membrane active layer thickness of 1 μm and using the appropriate permeance and solubility from this table

Table S3. Permeance, solubility and diffusivity of O_2 in the polyimide-based membrane from UBE UMS-A2 module.

308 K				318 K				328 K			
p[1]	Q[2]	S[3]	D[4]	p[1]	Q[2]	S[3]	D[4]	p[1]	Q[2]	S[3]	D[4]
1.69	$1.22\cdot10^{-2}$	1.02	$1.20\cdot10^{-6}$	1.72	$1.57\cdot10^{-2}$	0.84	$1.87\cdot10^{-6}$	1.70	$2.05\cdot10^{-2}$	0.70	$2.93\cdot10^{-6}$
2.37	$1.29\cdot10^{-2}$	1.00	$1.29\cdot10^{-6}$	2.39	$1.66\cdot10^{-2}$	0.83	$2.01\cdot10^{-6}$	2.04	$2.11\cdot10^{-2}$	0.69	$3.04\cdot10^{-6}$
3.05	$1.32\cdot10^{-2}$	0.98	$1.36\cdot10^{-6}$	3.05	$1.71\cdot10^{-2}$	0.81	$2.11\cdot10^{-6}$	2.41	$2.13\cdot10^{-2}$	0.69	$3.09\cdot10^{-6}$
3.72	$1.36\cdot10^{-2}$	0.96	$1.42\cdot10^{-6}$	3.73	$1.76\cdot10^{-2}$	0.80	$2.21\cdot10^{-6}$	2.72	$2.17\cdot10^{-2}$	0.68	$3.17\cdot10^{-6}$
4.45	$1.37\cdot10^{-2}$	0.94	$1.46\cdot10^{-6}$	4.42	$1.79\cdot10^{-2}$	0.78	$2.28\cdot10^{-6}$	3.07	$2.19\cdot10^{-2}$	0.68	$3.24\cdot10^{-6}$
5.13	$1.43\cdot10^{-2}$	0.92	$1.56\cdot10^{-6}$	5.11	$1.83\cdot10^{-2}$	0.77	$2.37\cdot10^{-6}$	3.42	$2.21\cdot10^{-2}$	0.67	$3.29\cdot10^{-6}$
5.11	$1.43\cdot10^{-2}$	0.92	$1.56\cdot10^{-6}$	5.13	$1.82\cdot10^{-2}$	0.77	$2.36\cdot10^{-6}$	3.76	$2.24\cdot10^{-2}$	0.67	$3.34\cdot10^{-6}$
5.81	$1.47\cdot10^{-2}$	0.90	$1.63\cdot10^{-6}$	5.80	$1.85\cdot10^{-2}$	0.76	$2.44\cdot10^{-6}$	4.10	$2.26\cdot10^{-2}$	0.66	$3.40\cdot10^{-6}$
6.48	$1.49\cdot10^{-2}$	0.89	$1.69\cdot10^{-6}$	6.31	$1.85\cdot10^{-2}$	0.75	$2.47\cdot10^{-6}$	4.45	$2.28\cdot10^{-2}$	0.66	$3.45\cdot10^{-6}$
7.17	$1.51\cdot10^{-2}$	0.87	$1.74\cdot10^{-6}$					4.45	$2.27\cdot10^{-2}$	0.66	$3.45\cdot10^{-6}$
								4.79	$2.29\cdot10^{-2}$	0.65	$3.49\cdot10^{-6}$
								5.12	$2.30\cdot10^{-2}$	0.65	$3.53\cdot10^{-6}$

[1] p is in bar
[2] Q is in cm^3(STP) cm^{-2} s^{-1} bar^{-1}
[3] S is in cm^3(STP) cm^{-3}(membrane) bar^{-1}, calculated from the Dual Mode Sorption (DMS) model with minimized the squared solubility differences
[4] D is in cm^2 s^{-1}, calculated from the partial immobilization model, assuming the membrane active layer thickness of 1 μm and using the appropriate permeance and solubility from this table

Table S4. Permeance, solubility and diffusivity of N_2 in the polyimide-based membrane from UBE UMS-A2 module.

308 K				318 K				328 K			
**p**[1]	**Q**[2]	**S**[3]	**D**[4]	**p**[1]	**Q**[2]	**S**[3]	**D**[4]	**p**[1]	**Q**[2]	**S**[3]	**D**[4]
1.67	$1.11 \cdot 10^{-3}$	0.85	$1.30 \cdot 10^{-7}$	1.66	$1.78 \cdot 10^{-3}$	0.70	$2.55 \cdot 10^{-7}$	1.34	$2.29 \cdot 10^{-3}$	0.58	$3.93 \cdot 10^{-7}$
2.37	$1.37 \cdot 10^{-3}$	0.83	$1.66 \cdot 10^{-7}$	2.35	$2.03 \cdot 10^{-3}$	0.68	$2.97 \cdot 10^{-7}$	1.67	$2.93 \cdot 10^{-3}$	0.58	$5.07 \cdot 10^{-7}$
3.05	$1.45 \cdot 10^{-3}$	0.81	$1.79 \cdot 10^{-7}$	3.02	$2.18 \cdot 10^{-3}$	0.67	$3.24 \cdot 10^{-7}$	2.03	$3.12 \cdot 10^{-3}$	0.57	$5.43 \cdot 10^{-7}$
3.73	$1.52 \cdot 10^{-3}$	0.79	$1.92 \cdot 10^{-7}$	4.43	$2.25 \cdot 10^{-3}$	0.65	$3.47 \cdot 10^{-7}$	2.03	$3.11 \cdot 10^{-3}$	0.57	$5.42 \cdot 10^{-7}$
5.82	$1.59 \cdot 10^{-3}$	0.74	$2.13 \cdot 10^{-7}$	5.11	$2.33 \cdot 10^{-3}$	0.64	$3.66 \cdot 10^{-7}$	2.37	$3.20 \cdot 10^{-3}$	0.57	$5.62 \cdot 10^{-7}$
6.52	$1.66 \cdot 10^{-3}$	0.73	$2.27 \cdot 10^{-7}$	5.53	$2.37 \cdot 10^{-3}$	0.63	$3.76 \cdot 10^{-7}$	3.03	$3.19 \cdot 10^{-3}$	0.56	$5.69 \cdot 10^{-7}$
7.20	$1.70 \cdot 10^{-3}$	0.72	$2.37 \cdot 10^{-7}$					3.38	$3.19 \cdot 10^{-3}$	0.56	$5.74 \cdot 10^{-7}$
								3.73	$3.23 \cdot 10^{-3}$	0.55	$5.84 \cdot 10^{-7}$
								4.09	$3.25 \cdot 10^{-3}$	0.55	$5.94 \cdot 10^{-7}$

[1] p is in bar
[2] Q is in cm^3(STP) cm^{-2} s^{-1} bar^{-1}
[3] S is in cm^3(STP) cm^{-3}(membrane) bar^{-1}, calculated from the Dual Mode Sorption (DMS) model with minimized the squared solubility differences
[4] D is in cm^2 s^{-1}, calculated from the partial immobilization model, assuming the membrane active layer thickness of 1 μm and using the appropriate permeance and solubility from this table

Table S5. Summary of the solubility, diffusivity and selectivity of pure CO_2, CH_4, O_2 and N_2 at 308 K in the polyimide-based membrane from UBE UMS-A2 module.

	CO_2		CH_4		O_2		N_2		α[4]					
**p**[1]	**S**[2]	**D**[3]	**S**[2]	**D**[3]	**S**[2]	**D**[3]	**S**[2]	**D**[3]	**CO_2/CH_4**	**CO_2/O_2**	**CO_2/N_2**	**O_2/CH_4**	**N_2/CH_4**	**O_2/N_2**
3	10.24	$6.71 \cdot 10^{-7}$	2.61	$2.76 \cdot 10^{-8}$	0.98	$1.38 \cdot 10^{-6}$	0.81	$1.84 \cdot 10^{-7}$	95.7	5.1	46.0	18.8	2.1	9.0
4	8.67	$7.58 \cdot 10^{-7}$	2.37	$3.16 \cdot 10^{-8}$	0.95	$1.46 \cdot 10^{-6}$	0.78	$1.96 \cdot 10^{-7}$	87.7	4.7	42.9	18.5	2.0	9.0
5	7.66	$8.13 \cdot 10^{-7}$	2.18	$3.54 \cdot 10^{-8}$	0.92	$1.54 \cdot 10^{-6}$	0.76	$2.07 \cdot 10^{-7}$	80.6	4.4	39.6	18.4	2.0	9.0
6	6.96	$8.48 \cdot 10^{-7}$	2.03	$3.88 \cdot 10^{-8}$	0.90	$1.62 \cdot 10^{-6}$	0.74	$2.17 \cdot 10^{-7}$	74.8	4.1	36.7	18.4	2.0	9.0
7	6.44	$8.73 \cdot 10^{-7}$	1.91	$4.18 \cdot 10^{-8}$	0.87	$1.70 \cdot 10^{-6}$	0.72	$2.28 \cdot 10^{-7}$	70.3	3.8	34.2	18.6	2.1	9.0
8	6.04	$8.90 \cdot 10^{-7}$	1.81	$4.45 \cdot 10^{-8}$	0.85	$1.77 \cdot 10^{-6}$	0.70	$2.38 \cdot 10^{-7}$	66.7	3.6	32.1	18.8	2.1	9.0
9	5.72	$9.02 \cdot 10^{-7}$	1.72	$4.69 \cdot 10^{-8}$	0.83	$1.85 \cdot 10^{-6}$	0.69	$2.47 \cdot 10^{-7}$	63.9	3.4	30.4	19.0	2.1	9.1
10	5.47	$9.11 \cdot 10^{-7}$	1.65	$4.90 \cdot 10^{-8}$	0.81	$1.92 \cdot 10^{-6}$	0.67	$2.57 \cdot 10^{-7}$	61.7	3.2	28.9	19.4	2.1	9.1
11	5.25	$9.19 \cdot 10^{-7}$	1.58	$5.09 \cdot 10^{-8}$	0.80	$1.99 \cdot 10^{-6}$	0.66	$2.66 \cdot 10^{-7}$	59.9	3.0	27.6	19.7	2.2	9.1
12	5.07	$9.24 \cdot 10^{-7}$	1.53	$5.25 \cdot 10^{-8}$	0.78	$2.05 \cdot 10^{-6}$	0.64	$2.74 \cdot 10^{-7}$	58.5	2.9	26.5	20.0	2.2	9.1
13	4.92	$9.29 \cdot 10^{-7}$	1.48	$5.40 \cdot 10^{-8}$	0.77	$2.12 \cdot 10^{-6}$	0.63	$2.82 \cdot 10^{-7}$	57.4	2.8	25.6	20.4	2.2	9.1
14	4.79	$9.32 \cdot 10^{-7}$	1.43	$5.52 \cdot 10^{-8}$	0.75	$2.18 \cdot 10^{-6}$	0.62	$2.90 \cdot 10^{-7}$	56.5	2.7	24.8	20.8	2.3	9.1
15	4.67	$9.35 \cdot 10^{-7}$	1.39	$5.64 \cdot 10^{-8}$	0.74	$2.24 \cdot 10^{-6}$	0.61	$2.98 \cdot 10^{-7}$	55.7	2.6	24.1	21.1	2.3	9.1
16	4.57	$9.38 \cdot 10^{-7}$	1.35	$5.74 \cdot 10^{-8}$	0.73	$2.30 \cdot 10^{-6}$	0.60	$3.05 \cdot 10^{-7}$	55.1	2.6	23.4	21.5	2.4	9.1
17	4.48	$9.40 \cdot 10^{-7}$	1.32	$5.83 \cdot 10^{-8}$	0.72	$2.35 \cdot 10^{-6}$	0.59	$3.12 \cdot 10^{-7}$	54.6	2.5	22.9	21.8	2.4	9.1
18	4.40	$9.42 \cdot 10^{-7}$	1.29	$5.92 \cdot 10^{-8}$	0.70	$2.41 \cdot 10^{-6}$	0.58	$3.19 \cdot 10^{-7}$	54.2	2.4	22.4	22.2	2.4	9.2

[1] p is in bar
[2] S is in cm^3(STP) cm^{-3}(membrane) bar^{-1}, calculated at 308 K from the Dual Mode Sorption (DMS) model with minimized the squared solubility differences
[3] D is in cm^2 s^{-1}, calculated at 308 K, assuming the membrane active layer thickness of 1 μm
[4] α is $(S_i/S_j) \times (D_i/D_j)$

Table S6. Summary of the solubility, diffusivity and selectivity of pure CO_2, CH_4, O_2 and N_2 at 318 K in the polyimide-based membrane from UBE UMS-A2 module.

	CO_2		CH_4		O_2		N_2		α[4]					
**p**[1]	**S**[2]	**D**[3]	**S**[2]	**D**[3]	**S**[2]	**D**[3]	**S**[2]	**D**[3]	CO_2/CH_4	CO_2/O_2	CO_2/N_2	O_2/CH_4	N_2/CH_4	O_2/N_2
3	8.61	$9.07 \cdot 10^{-7}$	2.15	$5.14 \cdot 10^{-8}$	0.81	$2.13 \cdot 10^{-6}$	0.67	$3.25 \cdot 10^{-7}$	70.8	4.5	35.7	15.7	2.0	7.9
4	7.36	$1.04 \cdot 10^{-6}$	1.98	$5.78 \cdot 10^{-8}$	0.79	$2.23 \cdot 10^{-6}$	0.65	$3.42 \cdot 10^{-7}$	66.6	4.3	34.1	15.5	2.0	7.9
5	6.54	$1.12 \cdot 10^{-6}$	1.85	$6.37 \cdot 10^{-8}$	0.77	$2.34 \cdot 10^{-6}$	0.64	$3.58 \cdot 10^{-7}$	62.3	4.1	32.0	15.4	1.9	7.9
6	5.96	$1.18 \cdot 10^{-6}$	1.73	$6.92 \cdot 10^{-8}$	0.76	$2.44 \cdot 10^{-6}$	0.62	$3.74 \cdot 10^{-7}$	58.4	3.8	30.1	15.3	1.9	7.9
7	5.53	$1.22 \cdot 10^{-6}$	1.64	$7.42 \cdot 10^{-8}$	0.74	$2.54 \cdot 10^{-6}$	0.61	$3.89 \cdot 10^{-7}$	55.2	3.6	28.3	15.4	1.9	7.9
8	5.19	$1.24 \cdot 10^{-6}$	1.56	$7.87 \cdot 10^{-8}$	0.72	$2.63 \cdot 10^{-6}$	0.60	$4.04 \cdot 10^{-7}$	52.6	3.4	26.8	15.5	2.0	7.9
9	4.93	$1.27 \cdot 10^{-6}$	1.49	$8.27 \cdot 10^{-8}$	0.71	$2.73 \cdot 10^{-6}$	0.58	$4.19 \cdot 10^{-7}$	50.4	3.2	25.5	15.7	2.0	7.9
10	4.71	$1.28 \cdot 10^{-6}$	1.44	$8.64 \cdot 10^{-8}$	0.70	$2.82 \cdot 10^{-6}$	0.57	$4.32 \cdot 10^{-7}$	48.7	3.1	24.3	15.8	2.0	7.9
11	4.53	$1.29 \cdot 10^{-6}$	1.38	$8.97 \cdot 10^{-8}$	0.68	$2.91 \cdot 10^{-6}$	0.56	$4.46 \cdot 10^{-7}$	47.3	2.9	23.3	16.0	2.0	7.9
12	4.38	$1.30 \cdot 10^{-6}$	1.34	$9.26 \cdot 10^{-8}$	0.67	$3.00 \cdot 10^{-6}$	0.55	$4.59 \cdot 10^{-7}$	46.1	2.8	22.5	16.3	2.0	7.9
13	4.25	$1.31 \cdot 10^{-6}$	1.30	$9.52 \cdot 10^{-8}$	0.66	$3.08 \cdot 10^{-6}$	0.54	$4.72 \cdot 10^{-7}$	45.1	2.7	21.7	16.5	2.1	7.9
14	4.14	$1.32 \cdot 10^{-6}$	1.26	$9.76 \cdot 10^{-8}$	0.65	$3.17 \cdot 10^{-6}$	0.53	$4.84 \cdot 10^{-7}$	44.3	2.7	21.1	16.7	2.1	7.9
15	4.04	$1.32 \cdot 10^{-6}$	1.23	$9.98 \cdot 10^{-8}$	0.64	$3.25 \cdot 10^{-6}$	0.53	$4.95 \cdot 10^{-7}$	43.7	2.6	20.5	17.0	2.1	8.0
16	3.95	$1.33 \cdot 10^{-6}$	1.20	$1.02 \cdot 10^{-7}$	0.63	$3.32 \cdot 10^{-6}$	0.52	$5.07 \cdot 10^{-7}$	43.1	2.5	20.0	17.2	2.2	8.0
17	3.87	$1.33 \cdot 10^{-6}$	1.17	$1.03 \cdot 10^{-7}$	0.62	$3.40 \cdot 10^{-6}$	0.51	$5.17 \cdot 10^{-7}$	42.6	2.4	19.5	17.4	2.2	8.0
18	3.81	$1.33 \cdot 10^{-6}$	1.14	$1.05 \cdot 10^{-7}$	0.61	$3.47 \cdot 10^{-6}$	0.50	$5.28 \cdot 10^{-7}$	42.3	2.4	19.1	17.7	2.2	8.0

[1] p is in bar
[2] S is in cm^3(STP) cm^{-3}(membrane) bar^{-1}, calculated at 318 K from the Dual Mode Sorption (DMS) model with minimized the squared solubility differences
[3] D is in cm^2 s^{-1}, calculated at 318 K, assuming the membrane active layer thickness of 1 μm
[4] α is $(S_i / S_j) \times (D_i / D_j)$

Table S7. Summary of the solubility, diffusivity and selectivity of pure CO_2, CH_4, O_2 and N_2 at 328 K in the polyimide-based membrane from UBE UMS-A2 module.

	CO_2		CH_4		O_2		N_2		α[4]					
**p**[1]	**S**[2]	**D**[3]	**S**[2]	**D**[3]	**S**[2]	**D**[3]	**S**[2]	**D**[3]	CO_2/CH_4	CO_2/O_2	CO_2/N_2	O_2/CH_4	N_2/CH_4	O_2/N_2
3	7.14	$1.25\cdot10^{-6}$	1.77	$8.94\cdot10^{-8}$	0.68	$3.23\cdot10^{-6}$	0.56	$5.65\cdot10^{-7}$	56.3	4.1	28.1	13.9	2.0	6.9
4	6.16	$1.43\cdot10^{-6}$	1.65	$1.00\cdot10^{-7}$	0.67	$3.37\cdot10^{-6}$	0.55	$5.89\cdot10^{-7}$	53.2	3.9	27.3	13.5	2.0	6.9
5	5.52	$1.55\cdot10^{-6}$	1.55	$1.11\cdot10^{-7}$	0.65	$3.50\cdot10^{-6}$	0.54	$6.13\cdot10^{-7}$	49.9	3.8	26.0	13.3	1.9	6.9
6	5.05	$1.64\cdot10^{-6}$	1.47	$1.20\cdot10^{-7}$	0.64	$3.63\cdot10^{-6}$	0.53	$6.36\cdot10^{-7}$	46.9	3.6	24.7	13.1	1.9	6.9
7	4.70	$1.70\cdot10^{-6}$	1.40	$1.29\cdot10^{-7}$	0.63	$3.75\cdot10^{-6}$	0.52	$6.58\cdot10^{-7}$	44.3	3.4	23.5	13.0	1.9	6.9
8	4.43	$1.75\cdot10^{-6}$	1.34	$1.37\cdot10^{-7}$	0.62	$3.88\cdot10^{-6}$	0.51	$6.80\cdot10^{-7}$	42.1	3.2	22.4	13.0	1.9	6.9
9	4.21	$1.78\cdot10^{-6}$	1.29	$1.44\cdot10^{-7}$	0.61	$4.00\cdot10^{-6}$	0.50	$7.01\cdot10^{-7}$	40.4	3.1	21.5	13.0	1.9	6.9
10	4.04	$1.81\cdot10^{-6}$	1.24	$1.51\cdot10^{-7}$	0.60	$4.12\cdot10^{-6}$	0.49	$7.21\cdot10^{-7}$	38.9	3.0	20.6	13.1	1.9	6.9
11	3.89	$1.83\cdot10^{-6}$	1.20	$1.57\cdot10^{-7}$	0.59	$4.23\cdot10^{-6}$	0.48	$7.41\cdot10^{-7}$	37.6	2.9	19.9	13.2	1.9	6.9
12	3.76	$1.84\cdot10^{-6}$	1.17	$1.62\cdot10^{-7}$	0.58	$4.34\cdot10^{-6}$	0.48	$7.60\cdot10^{-7}$	36.6	2.8	19.2	13.2	1.9	6.9
13	3.66	$1.85\cdot10^{-6}$	1.13	$1.67\cdot10^{-7}$	0.57	$4.45\cdot10^{-6}$	0.47	$7.79\cdot10^{-7}$	35.7	2.7	18.6	13.4	1.9	6.9
14	3.56	$1.86\cdot10^{-6}$	1.11	$1.72\cdot10^{-7}$	0.56	$4.56\cdot10^{-6}$	0.46	$7.97\cdot10^{-7}$	35.0	2.6	18.1	13.5	1.9	7.0
15	3.48	$1.87\cdot10^{-6}$	1.08	$1.76\cdot10^{-7}$	0.55	$4.66\cdot10^{-6}$	0.46	$8.14\cdot10^{-7}$	34.4	2.5	17.6	13.6	2.0	7.0
16	3.41	$1.88\cdot10^{-6}$	1.05	$1.80\cdot10^{-7}$	0.55	$4.76\cdot10^{-6}$	0.45	$8.31\cdot10^{-7}$	33.8	2.5	17.2	13.7	2.0	7.0
17	3.34	$1.89\cdot10^{-6}$	1.03	$1.83\cdot10^{-7}$	0.54	$4.86\cdot10^{-6}$	0.44	$8.47\cdot10^{-7}$	33.4	2.4	16.8	13.9	2.0	7.0
18	3.29	$1.89\cdot10^{-6}$	1.01	$1.86\cdot10^{-7}$	0.53	$4.96\cdot10^{-6}$	0.44	$8.63\cdot10^{-7}$	33.0	2.4	16.5	14.0	2.0	7.0

[1] p is in bar
[2] S is in cm^3(STP) cm^{-3}(membrane) bar^{-1}, calculated at 328 K from the Dual Mode Sorption (DMS) model with minimized the squared solubility differences
[3] D is in cm^2 s^{-1}, calculated at 328 K, assuming the membrane active layer thickness of 1 μm
[4] α is $(S_i/S_j) \times (D_i/D_j)$

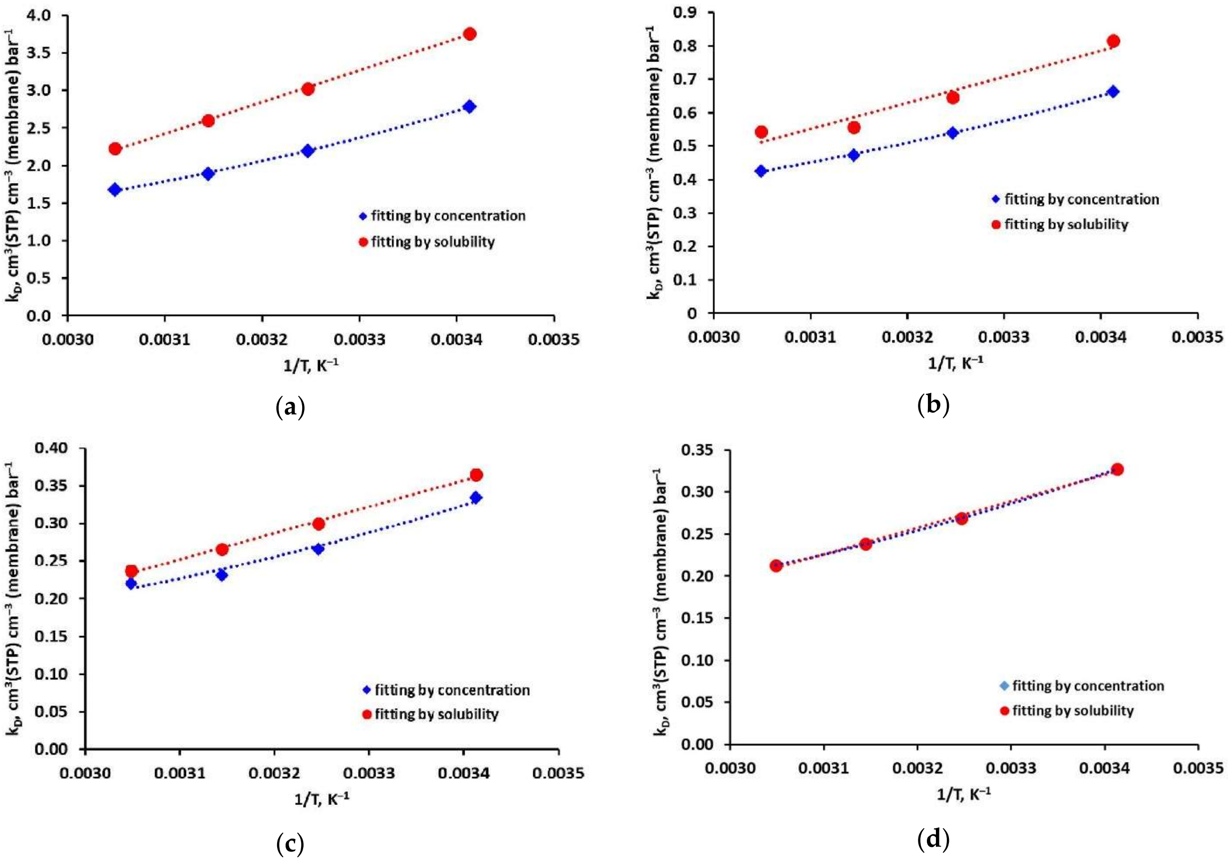

Figure S1. Temperature dependence of the Henry's constant in the Dual Mode Sorption (DMS) model for (**a**) CO_2, (**b**) CH_4, (**c**) O_2 and (**d**) N_2. The dotted lines are obtained from the fit.

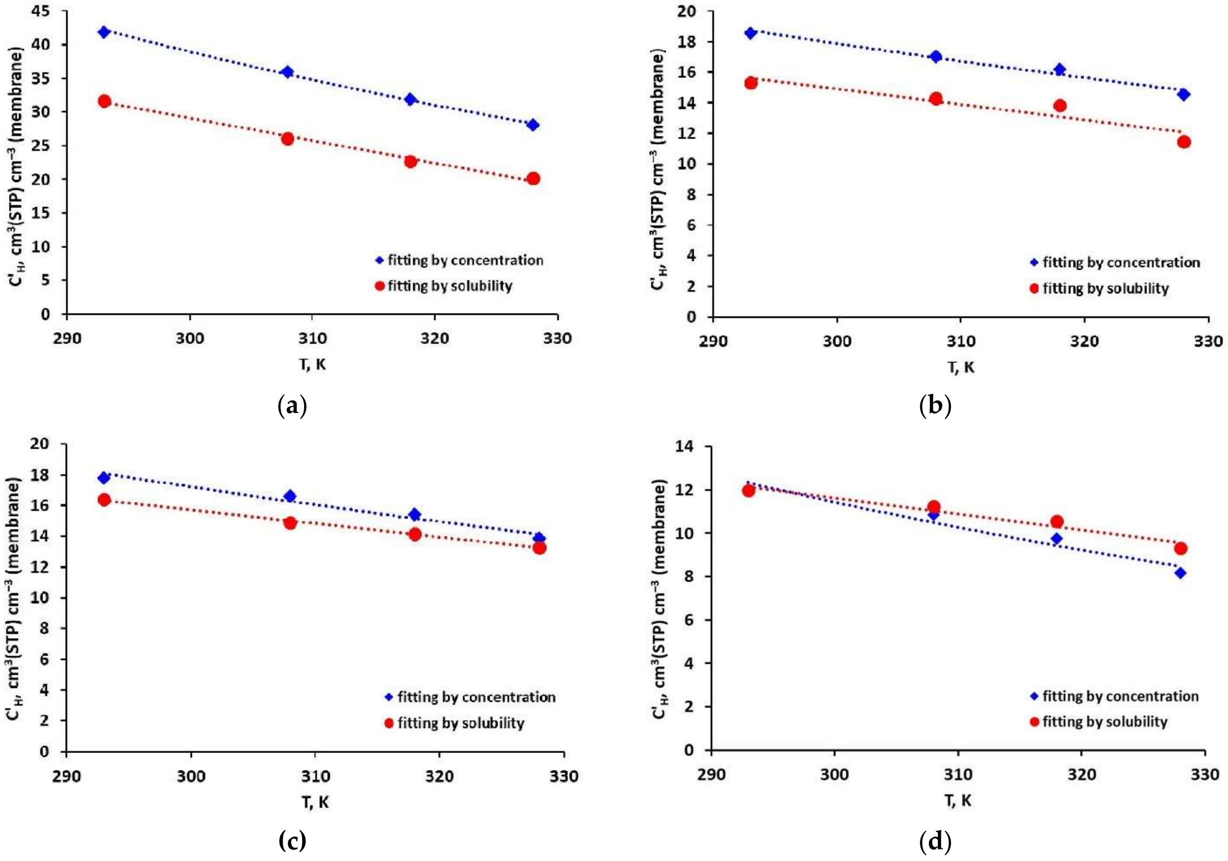

Figure S2. Temperature dependence of the Langmuir adsorption capacity in the Dual Mode Sorption (DMS) model for (**a**) CO_2, (**b**) CH_4, (**c**) O_2 and (**d**) N_2. The dotted lines are obtained from the fit.

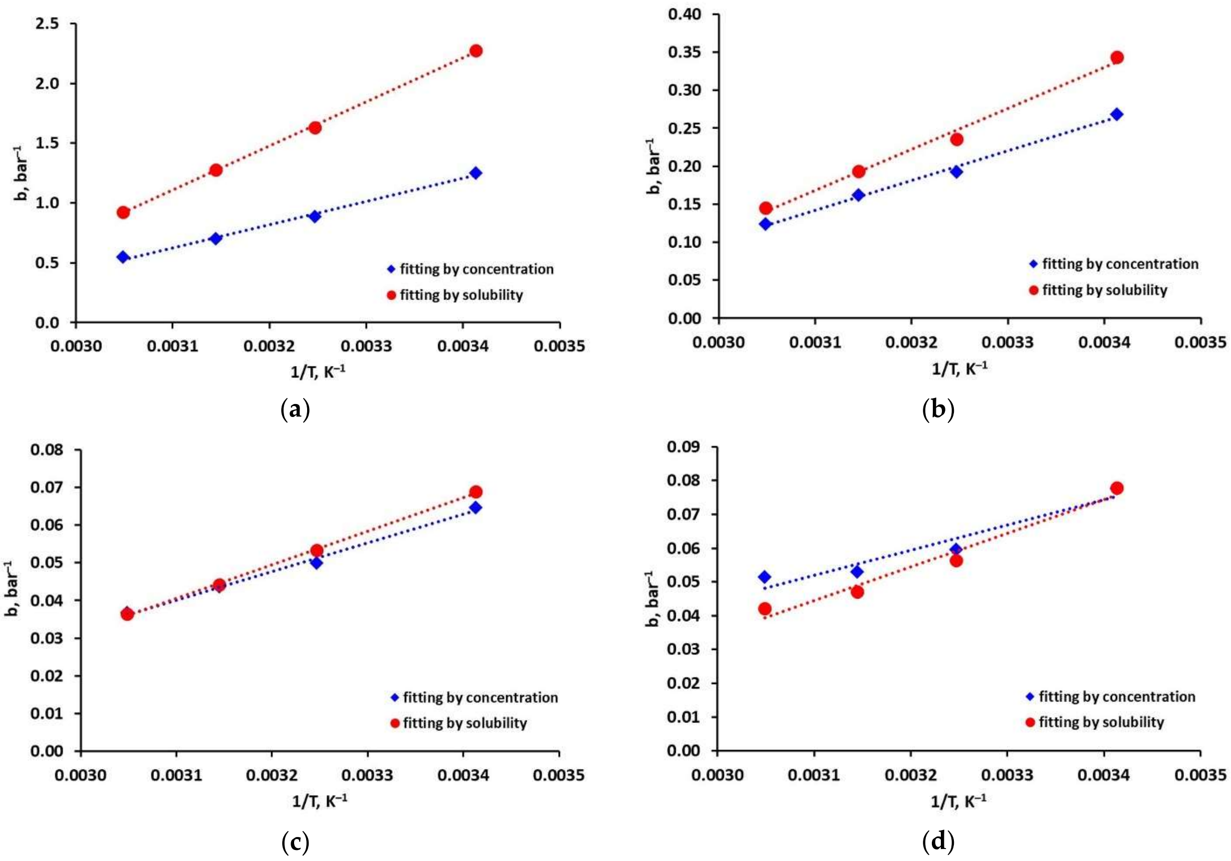

Figure S3. Temperature dependence of the Langmuir affinity constant in the Dual Mode Sorption (DMS) model for (**a**) CO_2, (**b**) CH_4, (**c**) O_2 and (**d**) N_2. The dotted lines are obtained from the fit.

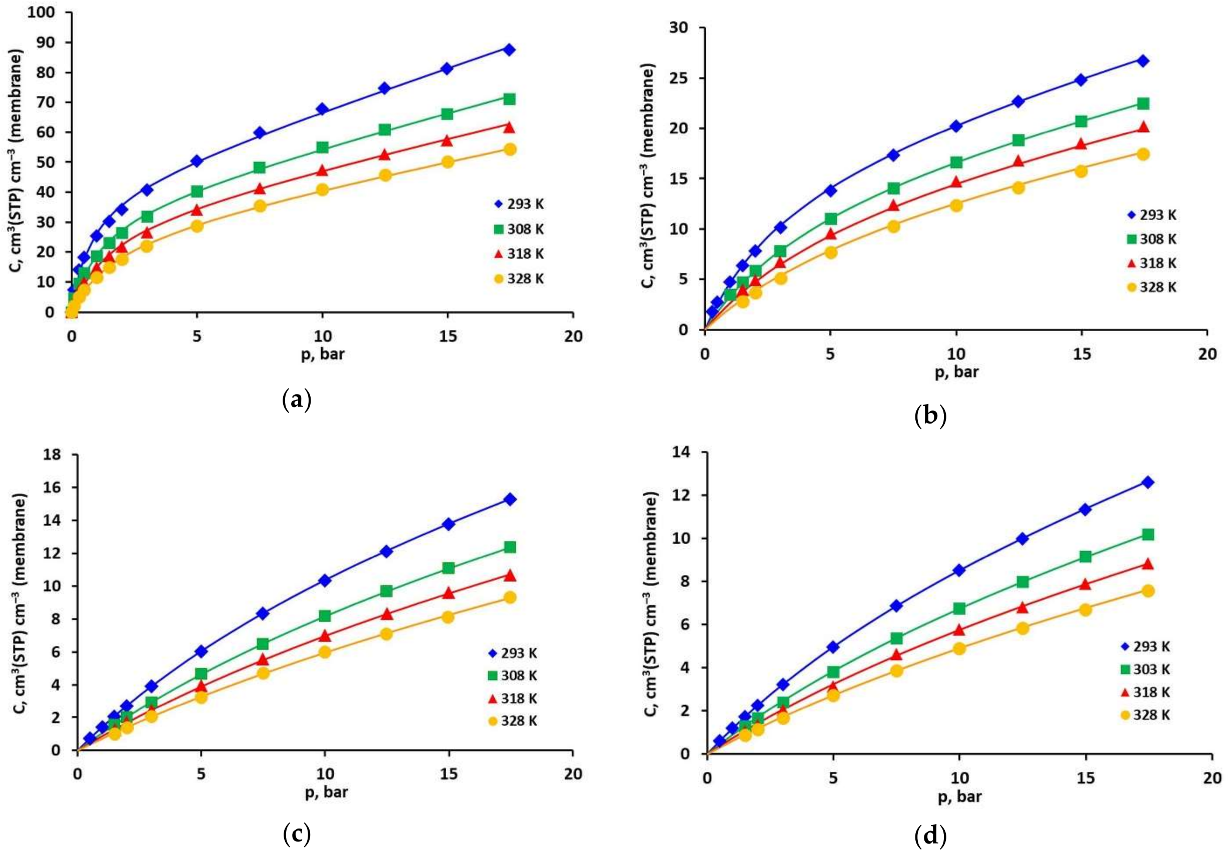

Figure S4. Concentration of pure (**a**) CO_2, (**b**) CH_4, (**c**) O_2 and (**d**) N_2 in the polyimide-based membrane from UBE UMS-A2 module. Points represent experimental data and lines Dual Mode Sorption (DMS) model predictions (for minimized the squared concentration differences).

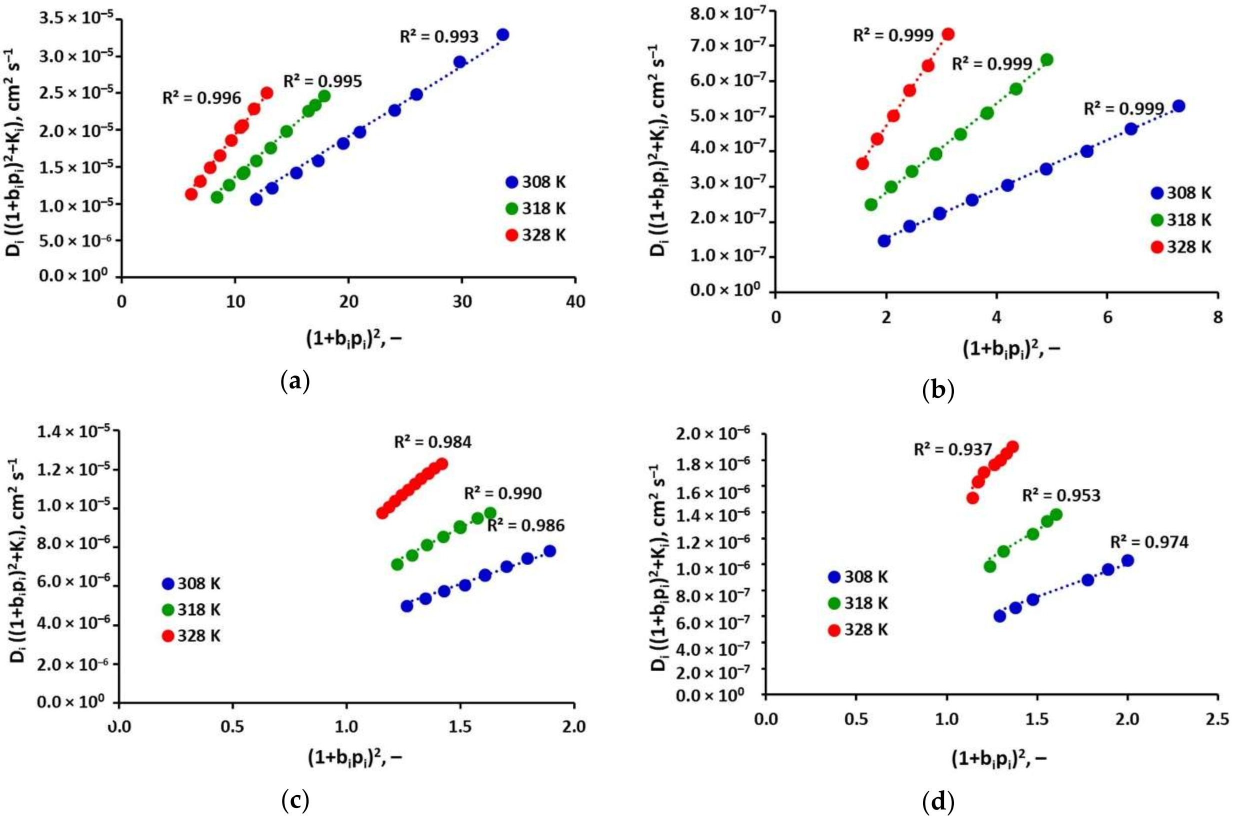

Figure S5. Diffusivity of pure (**a**) CO_2, (**b**) CH_4, (**c**) O_2 and (**d**) N_2 in the polyimide-based membrane from UBE UMS-A2 module according to the linearized partial immobilization model. A straight dotted line is from the fit. The case of the DMS model with minimized the squared solubility differences.
